# Non-apoptotic caspase events and Atf3 expression underlie direct neuronal differentiation of adult neural stem cells

**DOI:** 10.1242/dev.204381

**Published:** 2024-11-20

**Authors:** Frédéric Rosa, Nicolas Dray, Sébastien Bedu, Laure Bally-Cuif

**Affiliations:** Institut Pasteur, Université Paris Cité, CNRS UMR3738, Zebrafish Neurogenetics Unit, F-75015 Paris, France

**Keywords:** Adult neural stem cells, Telencephalon, Pallium, Zebrafish, Direct fate conversion, Apoptosis, Caspase, Atf3

## Abstract

Neural stem cells (NSCs) generate neurons over a lifetime in adult vertebrate brains. In the adult zebrafish pallium, NSCs persist long term through balanced fate decisions. These decisions include direct neuronal conversions, i.e. delamination and neurogenesis without a division. To characterize this process, we reanalyze intravital imaging data of adult pallial NSCs, and observe shared delamination dynamics between NSCs and committed neuronal progenitors. Searching for mechanisms predicting direct NSC conversions, we build an NSC-specific genetic tracer of Caspase3/7 activation (Cas3*/Cas7*) *in vivo*. We show that non-apoptotic Cas3*/7* events occur in adult NSCs and are biased towards lineage termination under physiological conditions, with a predominant generation of single neurons. We further identify the transcription factor Atf3 as necessary for this bias. Finally, we show that the Cas3*/7* pathway is engaged by NSCs upon parenchymal lesion and correlates with NSCs more prone to lineage termination and neuron formation. These results provide evidence for non-apoptotic caspase events occurring in vertebrate adult NSCs and link these events with the NSC fate decision of direct conversion, which is important for long-term NSC population homeostasis.

## Teaser

**Highlighted Article:** An inducible Cas3*/Cas7* sensor demonstrates that non-apoptotic caspase events occur in zebrafish neural stem cells. In cooperation with the stress-induced transcription factor Atf3, they often result in direct neuronal generation.

## INTRODUCTION

Stem cell (SC) fate decisions orchestrate adult organ maintenance and SC population renewal over a lifetime. In the brain and muscle, some adult SCs can directly acquire a differentiated fate in the absence of a division event under physiological conditions (Barbosa et al., 2015; Bjornson et al., 2012; Dray et al., 2015; Ismaeel et al., 2024; Mourikis et al., 2012). These events, described as ‘direct conversion’, have remained understudied, and it is largely unknown how they are controlled.

In the adult vertebrate brain, neural stem cells (NSCs) are radial glia-like progenitors (RGs), which are mostly quiescent (Chaker et al., 2016; Lampada and Taylor, 2023; Obernier and Alvarez-Buylla, 2019; Urbán et al., 2019; Yeh et al., 2023). In mouse, NSCs can generate differentiated neurons and astrocytes (Beckervordersandforth et al., 2010; Dulken et al., 2017; Zywitza et al., 2018). In zebrafish, NSCs express *glial fibrillary acidic protein* (*gfap*) and *her4* genes, and co-express markers of mature astrocytes during their quiescence phase (Cosacak et al., 2019; Morizet et al., 2024). Upon division, they generate other NSCs (*gfap*^pos^, *her4*^pos^) and/or committed neuronal progenitors (NPs) (*gfap*^neg^, *her4*^neg^) that differentiate into neurons (Dirian et al., 2014; Furlan et al., 2017; Kroehne et al., 2011; Mancini et al., 2023). In both models, genetic clonal tracing and/or intravital imaging also suggests direct conversions from individual NSCs to neurons. In the mouse dentate gyrus, around 10% of clones in *Nestin:Cre* tracing of individual NSCs are single neurons after 1 month (Bonaguidi et al., 2011), suggesting a direct conversion, although the interpretation of these results could be confounded by cell death. In the zebrafish pallium, intravital imaging has revealed direct neuronal differentiation occurring from *her4.3*^pos^ or *gfap*^pos^ NSCs that do not express proliferation markers and/or have not divided for at least 14 days (four imaging time points (tp), 14-16 days] (Barbosa et al., 2015; Dray et al., 2015; Than-Trong et al., 2020). Longitudinal imaging in these cases shows no cell death events. Modeling clonal dynamics predicts that direct conversions represent 25% of fate decisions (Than-Trong et al., 2020), and are crucial to maintain homeostasis of the NSC population.

As part of our efforts to identify predictors and mechanisms of NSC fate decisions *in vivo*, we focus here on NSC direct conversions. First, exploiting the amenability of the zebrafish adult pallium to intravital NSC imaging, we quantify the morphodynamic features of direct conversion events in real time. We show that these parameters do not differ from those of NP delaminations, precluding the simple identification of morphometric predictors of direct conversions. As a second approach, we took an educated guess and challenged whether direct conversions could relate to non-apoptotic caspase events.

Caspases are site-specific proteases of the programmed cell death system. In addition, they are increasingly recognized for functions in non-apoptotic events during development and homeostasis in various tissues and organisms (reviewed by Abdul-Ghani and Megeney, 2008; Burgon and Megeney, 2018). In particular, there is a frequent association of non-apoptotic caspase events with cell fate decisions, including in some SCs (Fujita et al., 2008; Janzen et al., 2008). In the nervous system, non-apoptotic caspase recruitments modulate dendritic pruning, developmental circuit maturation and axonal pathfinding (Unsain and Barker, 2015), neuronal differentiation (Fernando et al., 2005), and neural progenitor proliferation (Colon-Plaza and Su, 2022; Kuranaga et al., 2006). Non-apoptotic caspase events involve the activation of effector Caspases 3 and 7 (activated forms noted as Cas3*, Cas7*) by proteolytic cleavage, and can be tracked using Cas3*/Cas7* sensors. For example, in *Drosophila*, CasExpress and CaspaseTracker liberate Gal4 upon cleavage at the Cas3*/Cas7* canonical site DEVD, to drive lineage labeling when caspase activation is not followed by cell death (Ding et al., 2016; Tang et al., 2015). These studies revealed multiple cells surviving an early caspase event under physiological conditions. These events can be seen as true death reversals (referred to as anastasis, as in Sun et al., 2017; Tang and Tang, 2018) or sign common pathways used to trigger cell remodeling in apoptosis and cellular decisions.

To assay whether non-apoptotic caspase events take place in adult NSCs, and, if so, test their relationship with NSC direct conversion, we generated an inducible, NSC-specific and stable Cre-mediated Cas3*/Cas7* sensor, *Cas^CRE^Atlas*. We find that non-apoptotic caspase events do take place in NSCs and are biased towards fate choices of direct neuronal generation. We identify the stress-induced transcription factor Atf3 as necessary for this NSC fate *in vivo*. Finally, we show that NSCs undergoing Cas3*/Cas7* events are further prone to neurogenic fates in response to lesion.

## RESULTS

### Direct conversion events and post-division delaminations share morphodynamic features

Using intravital imaging, NSC direct conversions were previously defined as the loss of expression of the NSC marker *Tg(gfap:dTomato)* accompanied with delamination from the pallial ventricular layer without visible division during the preceding four imaging time points (4 tp) (14 to 16 days) (Than-Trong et al., 2020). This time frame was chosen because, when a neurogenic division is visible in a movie, 85±10% (mean±s.d.) of neuron-fated daughter cells visibly express their fate (loss of *gfap*:dTomato) within the 10-12 days post-division (Dray et al., 2021; Than-Trong et al., 2020). Such events are followed by expression of the neuronal differentiation marker HuC/D and the presence of a neuronal process (Barbosa et al., 2015).

**Fig. 1.**
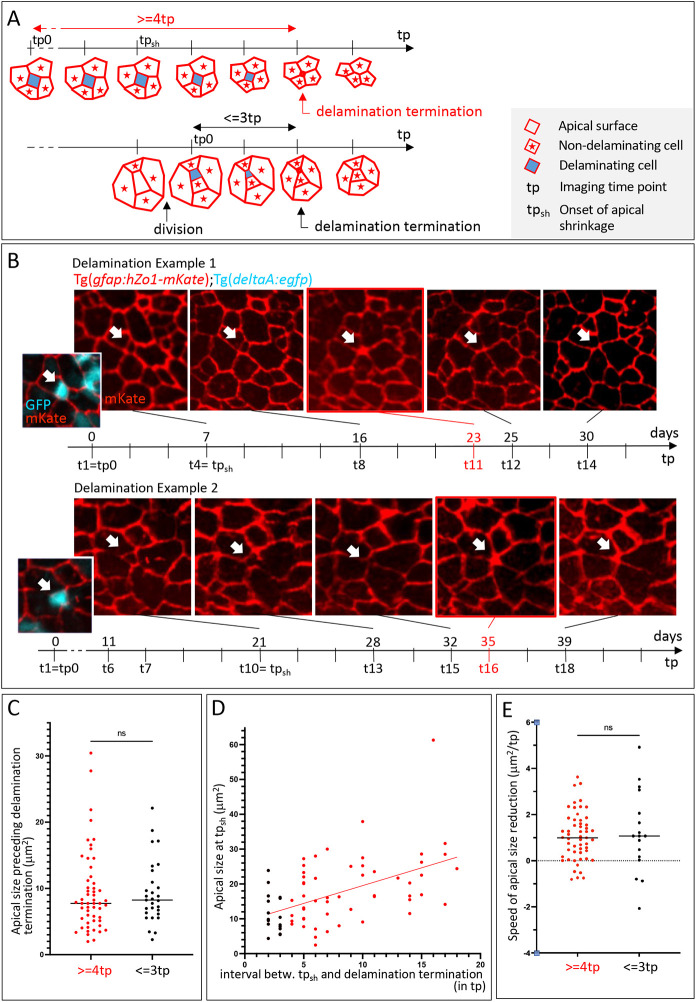
**Morphodynamic characterization of delamination events in the adult pallium.** (A) Schematics of delamination events observed using intravital imaging in *Tg(gfap:Zo1-mKate2)* 3 mpf adults (apical views). Horizontal arrows indicate imaging time points (tp); red indicates Zo1; blue indicates the apical surface of a delaminating cell; red stars indicate non-delaminating neighbors. Top: Tracking with ≥4 tp (14-16 days) without visible division prior to delamination termination. tp0, first tp of the movie; tp_sh_, apical size shrinkage onset. Bottom: Division event taking place ≤3 tp (≤13 days) before delamination termination. tp0, first time point post-division. (B) Snapshots of two delamination events recorded from a *Casper*;*Tg(gfap:hZo1-mKate)*;*Tg(deltaA:egfp)* 3 mpf adult (fish Outi; raw data taken from Mancini et al., 2023) (Zo1-mKate channel only, red). White arrows indicate delaminating cells; red frame highlights delamination termination; vertical time ticks indicate imaging tp (bottom) and corresponding days (top). Apical area of cells in examples 1 and 2: 20.1 µm2 and 22.9 µm2, respectively. Insets show images including the *deltaA*:eGFP channel (cyan) at shrinkage onset. (C-E) Quantified dynamic parameters of delaminations occurring >4 tp without division (red) and ≤3 tp post-division (black). (C) Apical surface area at tp preceding delamination termination. Mann–Whitney test, *P*=0.661. (D) Apical surface area at tp_sh_ as a function of the duration between tp_sh_ and delamination termination. Linear regression, R^2^=0.256. (E) Speed of apical size reduction calculated from tp_sh_ until delamination termination. Mann–Whitney test, *P*=0.849. ns, not significant.

To characterize the morphodynamic features of these events and determine whether and how they contrast with delamination and differentiation events occurring from neurogenic divisions (referred to as ‘post-division delaminations’), we exploited the intravital imaging movies acquired in 3 months post-fertilization (mpf) adults in the *Casper*;*Tg(gfap:Zo1-mKate2)*;*Tg(deltaA:egfp)* background (Mancini et al., 2023). This dataset contains 828 NSCs filmed over 39-43 days every 2-3 days in four pallial hemispheres (from four different fish) and reveals NSC apical surfaces (Zo1-mKate2) and a neurogenic fate (*deltaA* expression). This dataset includes 83 delamination events, which we now classified according to the 4 tp cut-off. When no division is visible along the track, tp0 is the first imaging tp of the movie (Fig. 1A, top), and we only consider delaminations taking place ≥4 tp from the start of each movie. When a division is visible, tp0 is the first tp post-division (Fig. 1A, bottom). We also defined ‘delamination termination’ as the first tp with no identifiable Zo1-mKate2-negative apical surface, i.e. the moment of apical closure (Fig. 1A). With these settings, 54 delaminations terminated after at least 4 tp (13 days) without visible division, and 29 followed a visible division during the previous three imaging tp or less (≤13 days). In the first category, the time from tp0 to delamination termination varied between 14 and 41 days. Two examples are illustrated in Fig. 1B, displaying delamination events that occurred without detectable division during the previous 23 (example 1) and 35 (example 2) days.

We next compared the morphometric parameters of delaminations occurring >4 tp without division versus ≤3 tp post-division (Fig. 1C-E). Cells in these two categories did not differ significantly in their apical area at the tp preceding delamination termination (9.4±6.1 µm^2^ and 9.3±4.8 µm^2^, respectively; mean±s.d.) (Fig. 1C), nor in their expression of *deltaA* (100% *deltaA^pos^* cells in both cases) (Fig. 1B). There was a moderate correlation between apical surface area at the onset of apical surface shrinkage (tp_sh_) and the duration of apical shrinkage (Fig. 1D). When normalized over the duration of shrinkage, shrinkage rates appeared similar between the two categories (∼1 µm^2^ per tp) (Fig. 1E). Together, these results characterize the quantitative and molecular features of the delamination process during adult pallial neurogenesis. They reveal its comparable dynamics irrespective of time post-division or apical surface area at the start.

The dataset does not allow NSCs and NPs to be directly recognized: NPs are *gfap:Zo1-mKate*^neg^, but, when intermingled among NSCs, they will appear as a Zo1-mKate-delineated surface, making them tractable. To estimate the percentage of NSCs in our dataset, we considered the two possible NP configurations: (1) NP clusters (e.g. an NP doublet generated by the symmetric neurogenic division of an NSC or an NP, which will appear as a single apical surface surrounded by ZO1-mKate), and (2) isolated NPs surrounded by NSCs. NP clusters can be quantified using immunohistochemistry (IHC) for Zo1 (also known as Tjp1), applied on specimen fixed at the end of the film, in comparison with mKate: NSCs are Zo1 IHC^pos^ and *gfap:Zo1-mKate*^pos^, while NPs are Zo1 IHC^pos^ and *gfap:Zo1-mKate*^neg^. Thus, we counted the proportion of Zo1-mKate surfaces that corresponded to several adjacent NPs (Fig. S1A). This proportion increased as the Zo1-mKate surface area decreased (Fig. S1B), as expected from NPs having smaller apical surface areas than NSCs (medians at 100 µm^2^ versus 20 µm^2^, respectively) (Mancini et al., 2023). With an apical surface area ≤30 µm^2^ at tp_sh_ (80/83 events, 96%) (see below and Fig. 1D), 21.4% of Zo1-mKate surfaces were NP clusters (Fig. S1B). Next, to count isolated NPs, an NSC marker was necessary. We used fixed *Tg(gfap:gfp)* pallia immunostained for GFP and Zo1; NSCs are *gfap:GFP*^pos^ and Zo1 IHC^pos^, while NPs are *gfap:GFP*^neg^ and Zo1 IHC^pos^ (Fig. S1C). With an apical surface area ≤30 µm^2^, 29.3% of surfaces were isolated NPs (Fig. S1D). Together, approximately half of the Zo1-mKate surfaces of 30 µm^2^ or less that would be recorded in intravital imaging were NSCs (Fig. S1E). Thus, the morphometric features measured above (Fig. 1) likely also apply to NSC direct conversions. Together, this analysis also shows that NSC direct conversions cannot be predicted using morphodynamic parameters alone.

### Non-apoptotic Cas3*/Cas7* events take place during pallium development and homeostasis

**Fig. 2.**
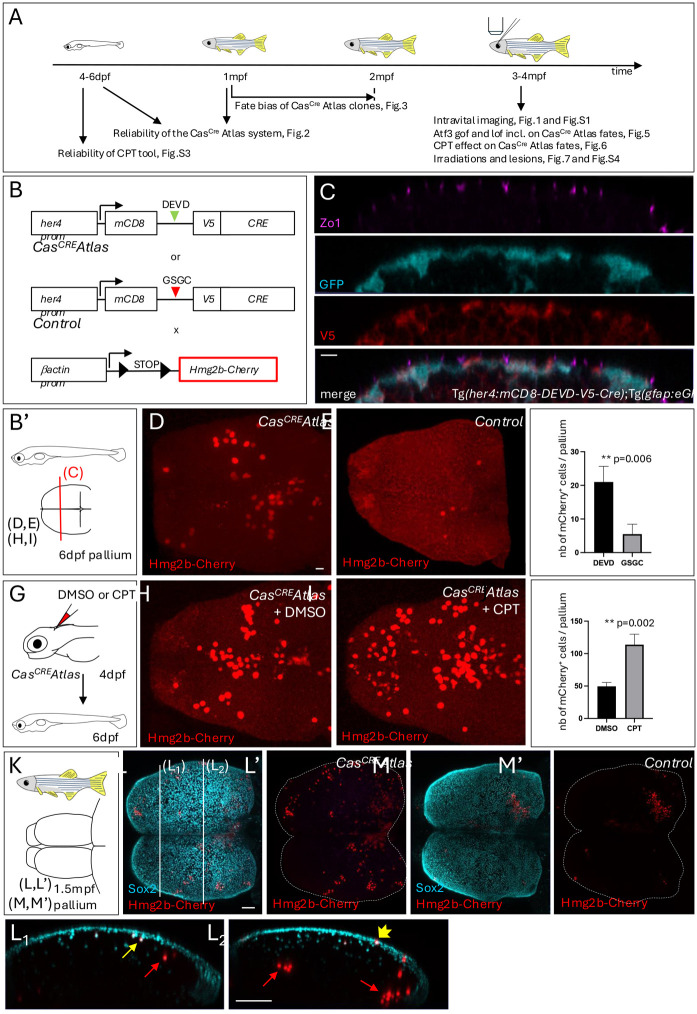
***Cas^CRE^Atlas* reveals non-apoptotic caspase events during pallium development and homeostasis.** (A) Experiments carried out in the present study shown in relation to the zebrafish lifetime. (B) Schematics of the transgenic lines used. *Cas^CRE^Atlas* combines an NP/NSC-specific driver line (top and middle) expressing Cre upon Cas3*/Cas7* cleavage and a reporter line (bottom) expressing *hmg2bmCherry* upon Cre recombination. mCD8, membrane anchor; V5, antigen segment (tag); DEVD, canonical Cas3*/Cas7* cleavage site; GSGC, non-productive cleavage site; *her4* prom, *her4* regulatory elements. The *Tg(her4:mCD8-DEVD-V5-Cre)*;*Tg(βact;lox-stop-lox-hmg2bmCherry)* background is referred to as *Cas^CRE^Atlas*, and *Tg(her4:mCD8-GSGC-V5-Cre)*;*Tg(βact;lox-stop-lox-hmg2bmCherry)* as ‘control’. All analyses were conducted in double heterozygotes. (B′) Schematic of analyses in cross-sectioned or whole-mount pallia at 6 dpf. (C) Expression of the *Cas^CRE^* driver is confined to *gfap:eGFP*-positive cells. Pallial cross-sections of *Tg(her4:mCD8-DEVD-V5-Cre)*;*Tg(gfap;eGFP)* larvae at 6 dpf with IHC for GFP (NP/NSC), Zo1 (tight junctions, delimiting apical surfaces) and V5 (transgene tag), showing V5 (membrane anchored) and GFP in the same cells. (D-F) Hmg2bmCherry whole-mount IHC on *Cas^CRE^Atlas* (D) and control (E) pallia at 6 dpf (dorsal views, *z*-projections, anterior left); quantification of positive cells (F) (*Cas^CRE^Atlas*: *n*=4 brains; control: *n*=10 brains; Mann–Whitney test). (G-J) *Cas^CRE^Atlas* activity is induced by camptothecin (CPT). (G) Schematic of the experiment: CPT (20 µM) or DMSO was injected into the neural tube ventricle in *Cas^CRE^Atlas* larvae at 4 dpf; Hmg2bmCherry was analyzed at 6 dpf. (H-J) Hmg2bmCherry whole-mount IHC at 6 dpf (dorsal views, *z*-projections, anterior left) and quantification (J) (DMSO and CPT: *n*=6 brains each; Mann–Whitney test). (K-M′) Hmg2bmCherry expression in *Cas^CRE^Atlas* (L,L′) and control (M,M′) pallia at 1.5 mpf. (K) Schematic of whole-mount pallia at 1.5 mpf. (L-M′) Whole-mount IHC for Sox2 (NPs, NSCs and some freshly born neurons) and Hmg2bmCherry (dorsal views, *z*-projections, anterior left). L_1_ and L_2_ show cross-sections as indicated in L, dorsal up. Red arrows indicate Sox2^neg^ neurons, thin yellow arrow Sox2^pos^ neurons, and short yellow arrow a Sox2^pos^ NSC. Scale bars: 10 µm (C-E,H,I); 50 µm (L-M′,L_1_,L_2_).

In search for predictive parameters of NSC direct conversions, we next considered non-apoptotic Caspase events (Fig. 2A). To track non-apoptotic proteolytic events at the DEVD Cas3*/Cas7* target site (Chéreau et al., 2003; Talanian et al., 1997), we designed a heritable lineage tracer of DEVD cleavage in NSCs. The driver line, *Tg(her4:mCD8-DEVD-V5-Cre)*, produces a Cre recombinase (fused to a V5 tag) tethered to the plasma membrane via a DEVD site, expressed under the *her4.3* regulatory elements (Yeo et al., 2007) (Fig. 2B). This promoter fragment drives expression in the same cells as the *gfap* regulatory elements, i.e. NSCs (Than-Trong et al., 2020). When *Tg(her4:mCD8-DEVD-V5-Cre)* fish are crossed into the *Tg(βact:lox-stop-lox-hmg2bmCherry)* reporter (Wang et al., 2011) (double background referred to as *Cas^CRE^Atlas*), non-apoptotic cleavage events at the DEVD site in *her4*-expressing cells should trigger Cre-mediated recombination and the permanent expression of Hmg2bmCherry in all progeny cells. A *Tg(her4:mCD8-GSGC-V5-Cre)* driver, immune to Cas3*/Cas7*, was used as control.

To validate the approach, we first analyzed the expression of the *her4:mCD8-DEVD-V5-Cre* transgene and the inducibility and selectivity of *Cas^CRE^Atlas* tracing in the larval pallium at 6 days post-fertilization (dpf). IHC in the *Tg(her4:mCD8-DEVD-V5-Cre)*;*Tg(gfap:eGFP)* background showed that V5-Cre expression is faithfully restricted to RGs (Fig. 2C). Next, we tested for Hmg2bmCherry^pos^ cells in the pallium at 6 dpf. Hmg2bmCherry^pos^ cells were rare in control pallia (on average, five per pallium). Their presence may reflect a low affinity of Cas3*/Cas7* for non-canonical sites, or the cleavage of GSGC by another protease at low frequency. In contrast, Hmg2bmCherry^pos^ cells were easily detectable and significantly more numerous in *Cas^CRE^Atlas* pallia (on average, 20 per pallium) (Fig. 2B′,D-F). We also tested whether *Cas^CRE^Atlas* was responsive to activation of the caspase cascade. To achieve this, 4 dpf larvae were transiently subjected to the apoptosis inducer camptothecin (CPT) (Ikegami et al., 1999) injected into the hindbrain ventricle, then chased until 6 dpf. CPT significantly increased the number of Hmg2bmCherry^pos^ cells in *Cas^CRE^Atlas* larvae compared to control injections (Fig. 2G-J).

The zebrafish pallium follows an outside-in neuronal generation pattern, without tangential neuronal migration (Furlan et al., 2017). We used these properties to interpret the pattern of Hmg2bmCherry cells in *Cas^CRE^Atlas* animals (Fig. 2K and see below). At juvenile and adult stages, neurogenesis follows the sequence NSCs (*her4*^pos^ or *gfap*^pos^; Sox2^pos^)>NPs (*her4*^neg^ and *gfap*^neg^; Sox2^pos^)>neurons (Than-Trong et al., 2020). At 1.5 mpf, few Hmg2bmCherry clones were seen in control pallia (Fig. 2M,M′). Based on their depth and position, they result from early non-specific activation events, as described at 6 dpf, generating rare but expanded clones. The Hmg2bmCherry pattern of *Cas^CRE^Atlas* pallia at 1.5 mpf was very different, with numerous small groups of labeled cells, some located in superficial layers (Fig. 2L,L′). IHC for Sox2, which also labels some recently born neurons, was used as a landmark and revealed deeply located (old) Sox2-positive neurons as well as more superficial (recent) events, with staining of freshly born neurons, NSCs or NPs (Fig. 2L_1_,L_2_). This pattern reflects Cre recombination events that occurred at different time points (from older to more recent) in *her4*-expressing cells.

### The Cas3*/Cas7*-driven lineage is biased towards neuronal differentiation

**Fig. 3.**
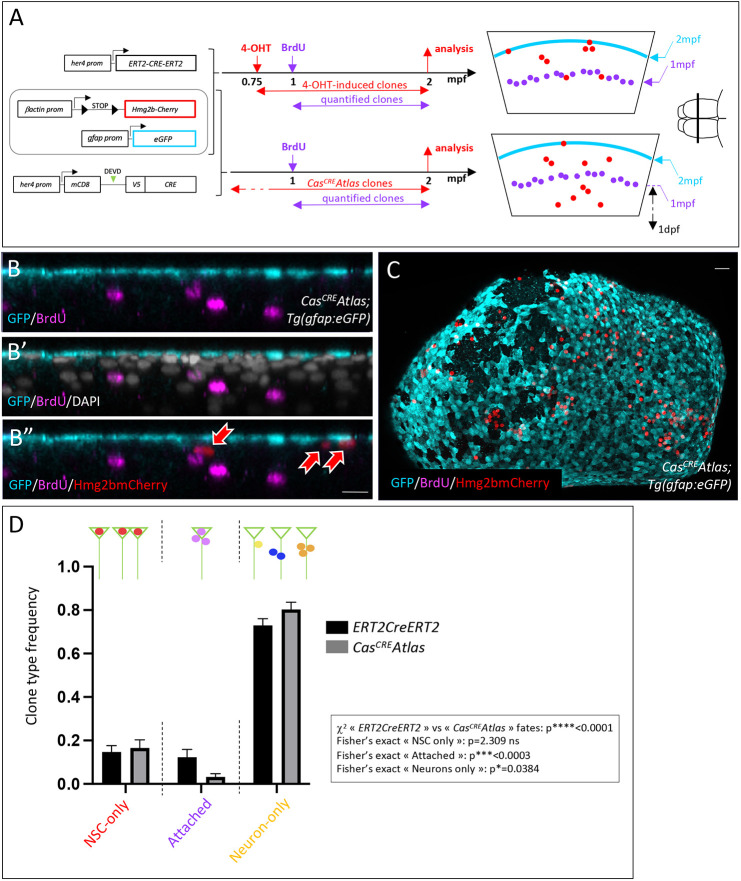
***Cas^CRE^Atlas* clones in pallial NSCs display a biased fate.** (A) *Tg(her4:ERT2CreERT2)*;*Tg(βact:lox-stop-lox-hmg2bmCherry)*;*Tg(gfap:eGFP)* (top) and *Cas^CRE^Atlas*;*Tg(gfap:eGFP)* (bottom) triple transgenic fish were used to compare NSC fates. Left: Genotypes. Middle: Schematic of the experiment. Right: Schematic cross-sections with relative positions of the NSC layer (cyan), BrdU-labeled neurons (magenta) and Hmg2bmCherry-positive clones (red) in each genotype. Schematic of the brain on the right (anterior left, dorsal view) shows the position of these schematic cross-sections. (B-B″) IHC for GFP (cyan, NSCs), BrdU (magenta) and Hmg2bmCherry (red) in a cross-section of a *Cas^CRE^Atlas*;*Tg(gfap:eGFP)* fish. Red arrows point to Hmg2bmCherry^pos^ cells. (C) Whole-mount *Cas^CRE^Atlas*;*Tg(gfap:eGFP)* 2mpf pallium with IHC for GFP, BrdU and Hmg2bmCherry (dorsal view, anterior left). (D) Frequency of the different Hmg2bmCherry^pos^ clone types (green triangles represent NSCs; colored dots represent Hmg2bmCherry^pos^ cells) generated from *her4*^pos^ NSCs between 1 and 2 mpf in the *her4:ERT2CreERT2* and *Cas^CRE^Atlas* backgrounds (black and gray bars, respectively) (*n*=5 brains for each; *her4:ERT2CreERT2* tracing: 344 clones; *Cas^CRE^Atlas*: 360 clones). Graph displays mean±s.e.m. Statistical analysis for global differences between *ERT2CreERT2* and *Cas^Cre^Atlas* fates: contingency χ^2^ test: ****P<0.0001. Statistical analyses for individual fates among three possible fates [‘NSC only’, ‘attached’, and ‘neurons only’ (one neuron or more)]: Fisher's exact test with Bonferroni correction: NSC only *P*=2.309; attached: ***P<0.0003; neurons only: *P=0.0384. ns, not significant. Scale bars: 15 µm (B,B′); 30 µm (C).

Cas3/Cas7 activation in *her4*^pos^ cells may, or not, correlate with specific NSC behaviors. To address this, we compared NSC fate in *Cas^CRE^Atlas* clones with *her4*-mediated NSC fate tracing in the juvenile to young adult pallium (Fig. 3A). We established a temporal landmark across pallial depth, using a bromodeoxyuridine (BrdU) pulse at 1 mpf, to label neurons born at that stage. Thus, *Cas^CRE^Atlas* events having occurred between 1 mpf and the stage of analysis (2 mpf) generated Hmg2bmCherry^pos^ cells located above the BrdU landmark (Fig. 3B-B″). These clones were categorized and quantified. In parallel, clonal recombination events were induced with 4-hydroxytamoxifen (4-OHT) in *Tg(her4:ERT2CreERT2)*; *Tg(βact:lox-stop-lox-eGFP)* animals just prior to the BrdU pulse, and the resulting clones were categorized and quantified at 2 mpf as well.

We considered that Hmg2bmCherry^pos^ cells belong to the same clones when they were separated by less than a two-cell diameter distance (Furlan et al., 2017; Webb et al., 2009), and recorded the composition of clones in 3D in whole-mount pallia using IHC for *gfap*:eGFP (NSCs). GFP^neg^ cells include NPs and neurons and, for simplicity, were labeled as neurons (Fig. 3C, Fig. S2A-A″). Unbiased clonal labeling generates a variety of clone types (Fig. 3D, Fig. S2B). ‘NSC-only’ clones are composed of single NSCs (remaining quiescent since labeling) or NSC doublets (from an amplifying NSC/NSC division) (red in Fig. S2A″). ‘Neuron-only’ clones include single neurons (presumably from a direct differentiation event), neuron doublets (from a direct differentiation event following an asymmetric neurogenic division, or from a symmetric neurogenic division), and groups of three neurons or more (from a direct differentiation or a symmetric neurogenic division following one or more neurogenic divisions) (respectively yellow, blue and orange in Fig. S2A″). Finally, ‘attached’ clones (magenta in Fig. S2A″) are made of an NSC and one or more neighboring neurons. This NSC was engaged in (a) recent neurogenic event(s), and attached clones correspond to neurogenically active NSCs. These fates together are in qualitative and quantitative agreement with the different division modes and fates reported in previous work (Than-Trong et al., 2020). On average, 50 *Cas^CRE^Atlas* events occurred during 4 weeks within a hemipallium (2000-2500 *her4*^pos^ NSCs). They were associated with most clone types; however, compared to *her4:ERT2CreERT2*-driven fates, attached clones were virtually absent in *Cas^CRE^Atlas* events, while the proportion of neuron-only clones was increased (Fig. 3D). Among the latter, the large majority (>75%) was made of single neurons (Fig. S2B). Thus, non-apoptotic Cas3*/Cas7* events in pallial NSCs correlate with a bias in the fate of neurogenically active NSCs towards lineage termination by neuronal differentiation.

### The Atf3 transcription factor is expressed in scattered delaminating cells at the adult pallial ventricle

**Fig. 4.**
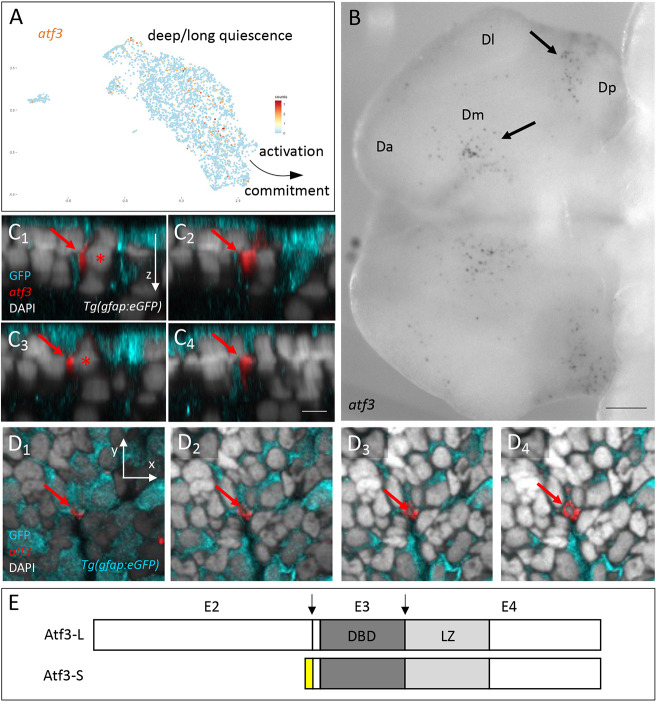
***atf3* is expressed in a subset of NSCs in the adult pallium.** (A) *atf3*^pos^ cells (orange dots, UMI values color-coded) on the scRNAseq UMAP of adult quiescent NSCs (blue dots). NSCs closest to activation and/or neurogenesis commitment and NSCs in deep/long quiescence are indicated (Morizet et al., 2024). (B) Expression of *atf3* in whole-mount ISH in the adult pallium (dorsal view, anterior left) with the *atf3-L* probe shown in E. Da, Dl, Dm, Dp, anterior, lateral, median and posterior pallial domains. Arrows point to *atf3*^pos^ cells in Dm and Dp. (C1-C4) Optical cross-sections of a *Tg(gfap:eGFP)* pallium stained in whole-mount for GFP (cyan, IHC), *atf3* transcripts (Fast Red) and DAPI (ventricular surface up). Sections are ordered from C1 to C4 along the anteroposterior axis. Red arrows indicate an *atf3*^pos^, GFP^pos^ cell (asterisk indicates the nucleus). (D1-D4) Optical horizontal sections of the pallium shown in C. D1 to D4 show superficial to deeper locations in sequence. Red arrows indicate an *atf3*^pos^, GFP^pos^ cell. (E) Atf3 protein isoforms predicted from adult pallial transcripts. DBD, DNA-binding domain; E2-E4, coding exons: L, long; LZ, leucine zipper; S, short. Arrows indicate splice junctions for the L form. Yellow box represents the N terminus of the S form (alternative splicing). Scale bars: 100 µm (B); 5 µm (C1-D4).

To identify the molecular events involved in promoting NSC direct differentiation, we searched among known mediators of non-apoptotic caspase events (Sun et al., 2017; Tang et al., 2017). Many genes upregulated at an early stage of anastasis appeared to be expressed in pallial NSCs under physiological conditions, as revealed in our previously generated single-cell RNA-sequencing (scRNAseq) dataset (Morizet et al., 2024) (Fig. S3A). We focused on *atf3*, one of the top genes induced in mammalian cells, expression of which was detectable in a low number of quiescent NSCs (Fig. 4A). Sparse expression was confirmed using whole-mount *in situ* hybridization (ISH), revealing scattered *atf3*^pos^ cells across the pallial surface (Fig. 4B), with some enrichment in the posterior and dorsomedial pallium. At small scale, the *atf3* pattern was not exactly identical in these areas in the two hemispheres. To address the location and morphology of *atf3*^pos^ cells, we used the *Tg(gfap:eGFP)* background and fluorescent chromogenic ISH. Cross-sections or horizontal optical sections showed that *atf3*^pos^;GFP^pos^ cells have a delaminating profile, with their nuclei often located in deeper positions than the majority of NSCs (Fig. 4C_1_-D_4_). We also compared *atf3* and Cas3. Of the two *casp3a* and -*b* duplicates, only *casp3a* expression was detected in scRNAseq (Fig. S3A), and only 5% of c*asp3a^pos^* cells were *atf3^pos^* in this dataset, equivalent to the overall proportion of *atf3^pos^* cells among all NSCs (Morizet et al., 2024). *In situ*, we only detected rare instances of co-expression of *atf3* and *casp3a* (using RNAScope Hiplex whole-mount ISH) (Fig. S3B) or *atf3* and Cas3* (revealed by IHC) under physiological conditions. Atf3 and Cas3* may be involved in distinct pathways, or at successive time points (or with very short windows of temporal overlap) in the same pathway, in adult pallial NSCs *in situ*. Finally, two *atf3* transcripts were recovered in the adult pallium, corresponding to alternative splicing events predicted to encode long (Atf3-L) and short (Atf3-S) protein isoforms that differ in their N terminus (Fig. 4E). Our ISH used the *atf3-L* probe and does not distinguish between these isoforms.

### Atf3 is necessary for the direct neuronal differentiation of adult pallial NSCs, and impacts physiological *Cas^CRE^Atlas* fate decisions

**Fig. 5.**
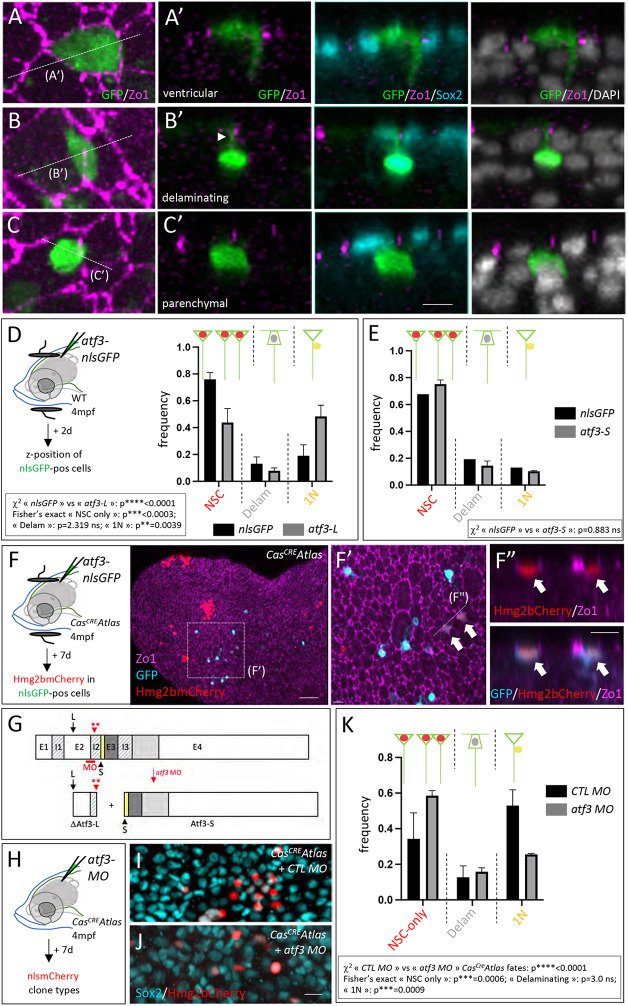
**Atf3 can drive NSC delamination and is needed for the *Cas^CRE^Atlas* fate bias.** (A-E) Cell fates expressed by adult pallial ventricular cells at 2 days post-electroporation (dpe). (A-C′) Visual categorization of the three possible fates in whole-mount pallia with IHC for GFP (green, electroporated construct), ZO1 (magenta) and Sox2 (cyan), and counterstaining with DAPI (gray). A-C: Dorsal (apical) views; A′-C′: Cross-sections as indicated in A-C. Arrowhead in B′ indicates apical attachment of a delaminating cell. (D,E) Experimental scheme and quantification of cell fate categories upon electroporation of *atf3-L-P2A-nlsGFP* versus *nlsGFP* (D) and *atf3-S-P2A-nlsGFP* versus *nlsGFP* (E). Graphs display mean±s.e.m. (D) *nlsGFP n*=214 cells, *atf3-L-P2A-nlsGFP n*=69 cells, from 4 and 3 hemipallia, respectively. Statistical analysis for global differences between *nlsGFP* and *atf3-L* fates: contingency χ^2^ test: ****P<0.0001; statistical analyses for individual fates among the three observed possible fates [NSCs (NSC singlets and doublets), single delaminating cells, and single neurons (1N)]: Fisher's exact test with Bonferroni correction: NSC ***P<0.0003; delaminating: *P*=2.319; neurons only: **P=0.0039. (E) *nlsGFP n*=62 cells, *atf3-S-P2A-nlsGFP n*=121 cells, from 2 and 3 hemipallia, respectively. Statistical analysis for global differences between *nlsGFP* and *atf3-S* fates: contingency χ^2^ test: *P*=0.883. (F-F″) Some NSCs overexpressing Atf3-F in *Cas^CRE^Atlas* double-transgenic adults are also Hmg2bmCherry^pos^ at 7 dpe. IHC for Zo1 (magenta), GFP (cyan, electroporated construct) and Hmg2bmCherry (red) in whole-mount pallium. F′ shows high magnification of the boxed area in F (dorsal views, anterior left); F″ shows magnified cross-section as indicated in F′. White arrows indicate double-positive cells. (G) Exon-intron structure of *atf3* (top) and predicted proteins (bottom) produced upon splice blockade by *atf3-MO* (red bar). E1-E4, exons; I1-I3, introns. Black arrows and arrowhead indicate ATG for Atf3-L (L) and Atf3-S (S), respectively; red arrowhead with double asterisk indicates double stop codon in I2. Color code as in Fig. 4E. (H-K) Atf3-L function blockade in *Cas^CRE^Atlas*. (H) Experimental scheme. (I,J) IHC for Hmg2bmCherry (red) and Sox2 (cyan) in whole-mount *Cas^CRE^Atlas* pallia 7 days after injection of the control MO (*CTL MO*; I) or *atf3-MO* (J) (dorsal views). (K) Quantification of the three observed *Cas^Cre^Atlas* clone types: ‘NSC-only’ (single NSCs and NSC doublets), ‘Delam’ (single delaminating cells) and single neurons (‘1N’). Graph displays mean±s.e.m. Statistical analysis for global differences between control MO and *atf3 MO*: contingency χ^2^ test: ****P<0.0001; statistical analyses for individual fates among the three observed possible fates: Fisher's exact test with Bonferroni correction: NSC ***P<0.0006; delaminating: *P*=3.0; 1N: ***P=0.0009. *n*=6 hemipallia per condition; control MO: *n*=82 clones; *atf3* MO: *n*=43 clones. ns, not significant. Scale bars: 8 µm (A-C); 50 µm (F,F′); 10 µm (F″); 8 µm (I,J).

Next, we combined gain- and loss-of-function experiments in adult pallial NSCs *in vivo* to test whether Atf3 impacts NSC fate. The ubiquitous *pCMV*:*nlsGFP* construct, electroporated after injection into the cerebral ventricle, highlights different cell fates on a short time scale [2 days post-electroporation (dpe)]: a large majority of ventricular cells (with a radial morphology and expressing Sox2, likely NSCs), and a minority of delaminating cells (with basally displaced nucleus and ventricular attachment, likely NPs) and of Sox2^neg^ parenchymal cells (interpreted as neurons) (Fig. 5A-C′). When electroporated under the same conditions, *pCMV:atf3-S-nlsGFP* had no effect on cell fate (Fig. 5E), while *pCMV:atf3-L-nlsGFP* significantly increased the proportion of neurons at the expense of NSCs (Fig. 5D). When electroporated into the *Cas^CRE^Atlas* background, *pCMV:atf3-L-nlsGFP* also correlated with Hmg2bmCherry expression in a minority of GFP^pos^ cells at 7 dpe (Fig. 5F-F″, white arrows). This may not result from chance, but the low occurrence of these co-expression events is puzzling. Cas3* may be induced only transiently or at levels too low to generate efficient levels of Cre recombinase. Alternatively, Atf3 may require a specific context to trigger Cas3* in adult NSCs. It is noteworthy that the hierarchical position of Atf3 relative to Cas3* has been reported to vary (see Discussion).

Next, to determine whether Atf3 is necessary for direct neuronal differentiation downstream of or in parallel to Cas3*, we tested the effect of blocking Atf3-L function *in vivo* in the *Cas^CRE^Atlas* double-transgenic context. We designed a vivo-morpholino (MO) directed against the exon2–intron2 boundary of *atf3*, predicted to generate a truncated Atf3-L protein devoid of its DNA-binding and leucine-zipper domains (Fig. 5G). This prediction was validated in embryos (Fig. S3C). This MO should not affect the production of the Atf3-S isoform, the start codon of which is located 3′ to the MO position. The *atf3* vivo-MO, or a control vivo-MO, were injected into the cerebral ventricle of *Cas^CRE^Atlas* adults, and *Cas^CRE^Atlas* fates were analyzed after 7 days (Fig. 5H-J). Hmg2bmCherry^pos^ clones were categorized when located within the first one or two cell rows below the ventricular surface (corresponding to the *z*-position of NSC progeny cells generated over a short time scale). We found that the *atf3* vivo-MO induced a significant increase in the proportion of clones composed of NSCs only, at the expense of the generation of single neurons (Fig. 5K). These results together indicate that Atf3-L expression is necessary for lineage bias, either downstream of or in parallel to physiological non-apoptotic Cas3* events.

### Experimentally induced Cas3*/Cas7* events drive direct neuronal production from adult NSCs

**Fig. 6.**
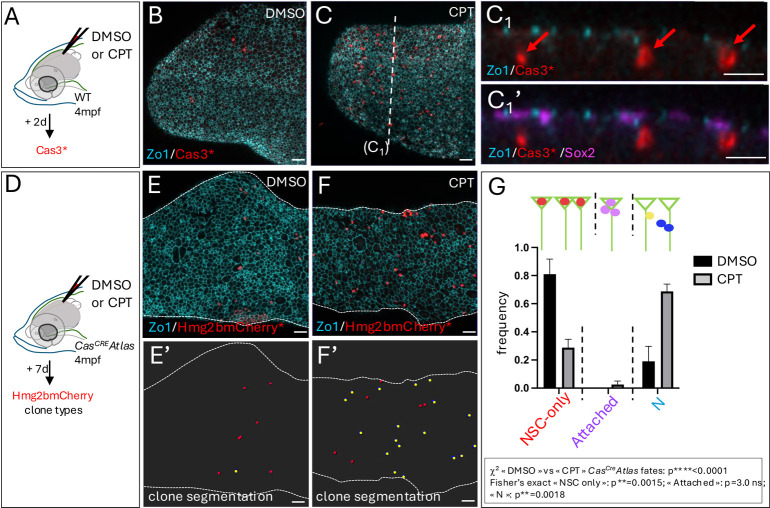
Experimentally induced Cas3* events drive direct neuronal differentiation in the adult pallium. (A-C_1_′) Induction of Cas3* by CPT (C) versus DMSO (B) injected into the cerebral ventricle (A), revealed by IHC in adult pallia at 2 days post-injection (dpi). (B,C) Dorsal whole-mount views (right hemisphere, anterior left) with IHC for Zo1 (cyan), Cas3* (red) and Sox2 (magenta, only shown in C_1_′). C_1_ and C_1_′ show cross-sections as indicated in B. Red arrows indicate delaminating Cas3*^pos^ cells. (D-G) Clone fate categories in *Cas^CRE^Atlas* adults upon injection of CPT (F,F′) versus DMSO (E,E′) at 7 dpi. (D) Experimental scheme. (E,F) Dorsal whole-mount views (right hemisphere, anterior left) processed for IHC for Zo1 (cyan) and Hmg2bmCherry* (red) for the DMSO (E) and CPT (F) treatments. (E′,F′) Segmentation of clones generated within 7 days (at and immediately below the pallial ventricular surface), color-coded [red, NSCs; yellow, parenchymal cells, identified as neurons (of which >87% were single neurons; no neuron doublets are visible on the illustrated fields)]. (G) Clone types at 7 days post-treatment. Graph displays mean±s.e.m. Statistical analysis for global differences between DMSO and CPT-induced *Cas^Cre^Atlas* fates: contingency χ^2^ test: ****P<0.0001; statistical analyses for individual fates among three possible fates [NSCs (NSC singlets and doublets), delaminating, and neurons only (1N or 2N)]: Fisher's exact test with Bonferroni correction: NSC **P=0.0015; attached: *P*=3.0; neurons only: **P=0.0018. Four hemipallia per condition. DMSO, *n*=37 clones; CPT *n*=89 clones. ns, not significant. Scale bars: 30 µm.

Cas3/Cas7 activation and Atf3-L appear, together, to be linked with the specific NSC fate choice of direct neuronal differentiation under physiological conditions. To address further the relevance of this regulatory process, we first tested whether experimental stimulation of Cas3/Cas7 activity in adult fish could modify NSC fate. CPT was used as an inducer. Incubation in CPT triggered Cas3* induction and was followed by NP death at larval stages [as revealed by the *Tg(ubi:secA5-mVenus)* reporter, whereby Annexin 5-mVenus expression marks cell death; van Ham et al., 2010] (Fig. S4A-B1). In striking contrast, pallial Cas3* cells were seen instead to delaminate when CPT was injected into the cerebral ventricle in adult animals (Fig. 6A-C_1_′). To track their fate longer term, CPT was applied to *Cas^CRE^Atlas* adults and clone types were assessed (Fig. 6D-G, Fig. S4C,D). We found a proportional increase of neuron-only clones (yellow in Fig. 6F′), mimicking the fate observed for physiological *Cas^CRE^Atlas* events in the adult pallium (Fig. 6G). Neuron-only clones were, in large majority (>70%), composed of single neurons (Fig. S4D). Over this short time frame, attached clones were virtually absent and the phenotype was observed at the expense of NSC clones (red in Fig. 6F′).

### Non-apoptotic Cas3* events are seldom recruited upon NSC irradiation but contribute to the generation of neurons during lesion repair

**Fig. 7.**
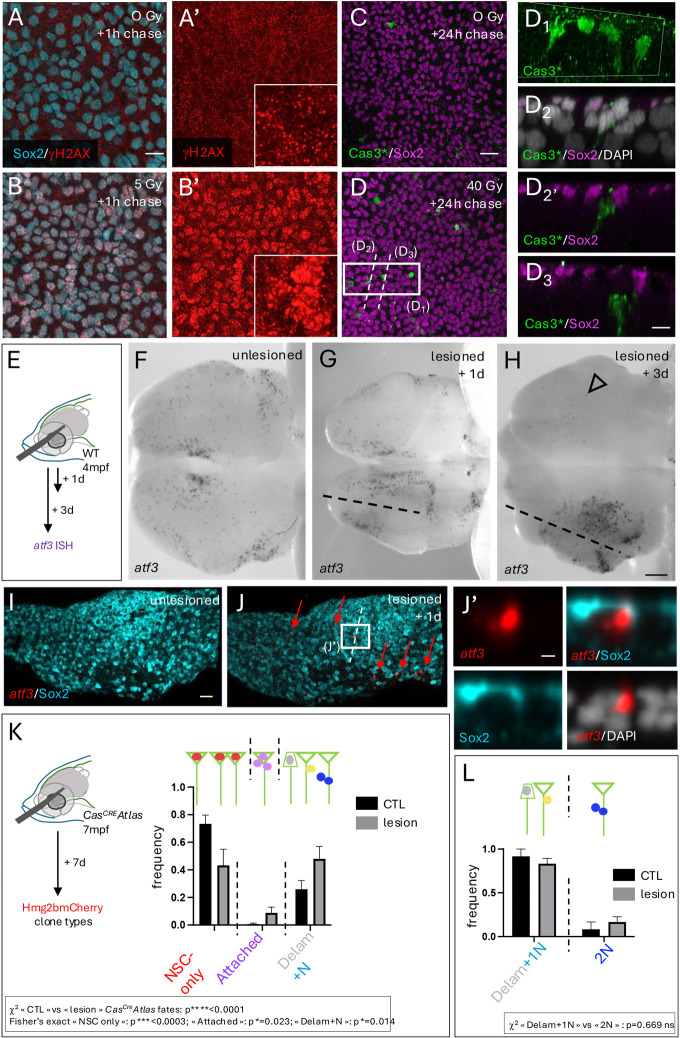
Differential recruitment of *atf3*^pos^ and Cas3* events under irradiation versus mechanical lesion. (A-D_3_) Effect of X-rays on NSCs *in vivo*. (A-B′) γH2AX DNA repair foci (red) in NSCs/NPs (Sox2^pos^, cyan) revealed by whole-mount IHC on adult pallia under control (A,A′) or irradiation conditions (B,B′) (5 Gy+1 h chase). (C-D_3_) Casp3* induction (green) in NSCs/NPs (Sox2^pos^, magenta) revealed by whole-mount IHC on adult pallia under control (C) or irradiation conditions (D) (40 Gy+24 h chase). D_1_ shows high magnification 3D rendering (Imaris) of the boxed area in D; D_2_, D_2_′ and D_3_ show sections across Cas3* cells as indicated in D. (E-L) Effect of mechanical lesion. (E-J′) *atf3* expression detected by chromogenic ISH (F-H: black; I-J′: red) on whole-mount pallia 1 day (G,I,J) or 3 days (H) following stab-wound injury (E) versus uninjured brains (F,I). Dorsal views, anterior left; one hemisphere is shown in I,J. Dashed lines indicate lesion trajectories; open arrowhead indicates downregulated *atf3* expression in the contralateral hemisphere at 3 dpl. (J′) High magnification section across an *atf3*^pos^ cell along the dashed line in J. (K) Proportion of *Cas^CRE^Atlas* clone types in control versus lesioned hemispheres at 7 dpl. Graph displays mean±s.e.m. Statistical analysis for global differences between *Cas^CRE^Atlas* clone types between lesioned and unlesioned hemispheres: contingency χ^2^ test: ****P<0.0001; statistical analyses for individual fates among three possible fates [NSCs (NSC singlets and doublets), attached clones, and neurogenic clones (delaminating cells, 1N or 2N; this category contains >70% 1N clones in both cases)]: Fisher's exact test with Bonferroni correction: NSC ***P<0.0003; attached: *P=0.023; Delam+N: *P=0.014. Control: 8 hemipallia, *n*=84 clones; Lesion: 7 hemipallia, 79 clones. (L) Proportion of neurogenic *Cas^CRE^Atlas* clone types in control versus lesioned hemispheres at 7 dpl, distinguishing single versus double neurons. Graph displays mean±s.e.m. Statistical analysis for global differences between single versus double neurons: contingency χ^2^ test: *P*=0.669. CTL, control; ns, not significant. Scale bars: 10 µm (A,A′,B,B′); 20 µm (C-C′); 10 µm (D1-D3); 100 µm (F-H); 30 µm (I,J); 10 µm (J′).

Finally, we tested whether Cas3*/Cas7* events were recruited under challenges that impact NSC state or fate. Quiescent NSCs are radiation resistant, possibly via an efficient mechanism of DNA repair (Barazzuol et al., 2019; Hellström et al., 2009; Mineyeva et al., 2019). However, radiation-induced differentiation has also been described (Konirova et al., 2019; Schneider et al., 2013). We used X-ray irradiation of live adults (3 mpf) to support this observation and test whether radiation resistance could also be accompanied by a NSC fate change *in vivo*. Short (1-h) treatment with a low radiation dose (5 Gy) induced γH2AX-positive foci in NSC/NP nuclei, indicative of double-strand DNA breaks and the recruitment of the repair machinery (Fig. 7A-B′). This process was complete by 2 h post-treatment (Fig. S5A-C″) with no visible effect on NSC fate and was observed until very high irradiation doses. At 40 Gy, a low number of cells located very close to the ventricular surface became Cas3*^pos^ after a 24-h chase (Fig. 7C-D_1_). These cells displayed a delaminating profile with a cell body partly displaced into the parenchyma (Fig. 7D_2_,D_3_) but sometimes keeping a ventricular attachment (Fig. 7D_2_-_2′_) and were Sox2^neg^. Considering that Cas3* induction may be rapid and transient (Andrews et al., 2016), we also conducted a minimal time series (9 h versus 24 h chase) with a 80 Gy dose, and observed a similar quantitative and qualitative phenotype at both time points (Fig. S5D-E′). Although we did not ascertain cell survival at later chase times, these results suggest that, upon irradiation at high dose, a few Cas3* events are induced and correlate with the first steps of neuronal commitment. Low to moderate irradiation schemes, however, lead to repair and do not recruit Cas3*.

Mechanical lesions applied to the adult pallium induce NSC division for neuronal repair (Baumgart et al., 2012; Kishimoto et al., 2012; Kroehne et al., 2011; März et al., 2011), accompanied with a partial fate shift towards symmetric neurogenic divisions (Barbosa et al., 2015). We tested whether *atf3*^pos^ or Cas3*/Cas7* events could also be involved in lesion response. *atf3* expression appeared to be induced around the lesioned ventricular zone starting at 1 day post-lesion (dpl) and very prominently at 3 dpl (Fig. 7E-H). It was also massively downregulated in the contralateral hemisphere at 3 dpl, a phenomenon not reported yet for other lesion-responsive genes (Fig. 7H, arrowhead). Like under physiological conditions (Fig. 4C1-D4), *atf3*^pos^ cells in lesioned pallia displayed a delaminating morphology (Fig. 7I-J′). We next used *Cas^CRE^Atlas* to track the fate of Cas3*/Cas7* events in a lesioned context. At 7 dpl, *Cas^CRE^Atlas* fates recorded in lesioned hemispheres were biased towards neurogenic fates at the expense of NSCs, compared to *Cas^CRE^Atlas* fates in non-lesioned brains (Fig. 7K). Among these neurogenic fates, in lesioned, like in unlesioned brains, the vast majority (>70%) corresponded to single neurons (Fig. 7L). We conclude that, within 7 dpl, the neurogenic fate of NSCs that experience a Cas3* event is further enhanced, without change in neurogenic mode. We have not attempted to link Atf3 and Cas3* in this context.

## DISCUSSION

In this work, we focus on adult NSC direct neuronal conversion and bring together several observations in link with this fate. First, we provide a quantitative, morphometric and molecular characterization of delamination/neuronal differentiation events in the NSC/NP population of the adult pallium. Second, we demonstrate that non-apoptotic Cas3*/Cas7* events occur in NSCs during development and homeostasis of the zebrafish pallium and, at adult stage, are preferentially associated with NSC fate choices of lineage termination by neuronal differentiation, among which the generation of single neurons is predominant. Upon parenchymal lesion or when induced by CPT, Cas3*/Cas7* events in NSCs are also further biased towards lineage termination and neuronal differentiation. Finally, we show that Atf3 is sufficient to trigger the direct generation of single neurons from NSCs and is necessary, downstream of or in parallel to Cas3*/Cas7*, for the realization of this fate. Together, our results provide a first molecular insight, with lineage and functional tracking *in vivo*, into NSC direct neuronal conversion. This fate decision balances fate choices to preserve homeostasis of the pallial NSC population over a lifetime (Than-Trong et al., 2020). Adding such a regulatory level beyond NSC division fate choices could add flexibility to the control of NSC numbers and neuronal production.

The *Tg(gfap:Zo1-mKate)*;*Tg(deltaA:egfp)* intravital imaging dataset includes NSCs and NPs that cannot be distinguished, but we estimate (Fig. S1) that around half of the recorded events are NSCs, and show that NSC conversions follow a classical delamination process. The observed significant duration of this process overall may permit important regulations of cell state towards neurogenesis during this event (Baek et al., 2018). The fact that delaminating NSCs do not undergo apoptosis and convert into neurons, not directly assessed here, is supported by arguments from other works: apoptosis is not observed in the adult pallium under normal conditions (Barbosa et al., 2015; Webb et al., 2009), neurons are the sole parenchymal fate of pallial NSCs (Furlan et al., 2017; März et al., 2010), and delaminating NSCs express neuronal markers (Barbosa et al., 2015).

Our analysis further suggests that *Cas^CRE^Atlas* captures a measurable fraction of these events, which are characterized by the expression of a specific molecular pathway involving Cas3*/Cas7* activation and Atf3. Several arguments support this conclusion. First, the direct conversion events recorded by intravital imaging (a few dozen events in ∼800 NSCs in 40 days) and the number of *Cas^CRE^Atlas* clones (37-46 events in ∼1000 NSCs in 56 days), the majority of which are single neurons, are of the same order or magnitude. Second, *Cas^CRE^Atlas* activation is tracked from NSCs and generates persisting clones (Fig. 3). Third, CPT treatment reveals that induction of the apoptosis cascade is sufficient to trigger NSC delamination (Fig. 6), while Atf3 blockade impairs the expression of the direct neuronal conversion fate (Fig. 5G-K), together suggesting that a cascade involving Cas3* and/or Cas7* and Atf3 actually drives the neuronal conversion fate. Obviously, intravital imaging of Cas3*/Cas7*/Atf3 events would be useful to connect these molecular events directly to our morphometric description, and this important aspect is currently lacking. This is, however, not possible to achieve with our current tools, notably because Hmg2B-mCherry needs several days to be directly visible by fluorescence, which would bypass the initial steps of the fate process. An NSC transcriptomic state possibly linked with direct conversions was recently proposed based on *in silico* analyses of scRNAseq data (Mitic et al., 2024). This interpretation remains to be validated with lineage and functional assays *in vivo*, but testing its relationship with Cas3*/Cas7* and Atf3 could be interesting.

The fact that non-apoptotic Cas3*/Cas7* events are physiological components of NSC population fates is an important finding: such events do occur in NSCs of the adult pallium and bias NSC fates towards lineage termination, including, in a large part, the direct generation of neurons (Fig. 3). To our knowledge, this is the first demonstration of non-apoptotic Cas3*/Cas7* events associated with a specific SC fate choice *in vivo*. Our work also provides information on the effectors or facilitators of this process. The cleavage site of the *Cas^CRE^Atlas* construct is also recognized by Cas7*. *casp7* is not expressed in the larval brain (Spead et al., 2018), arguing for a Cas3*-specific cleavage at larval stages (Fig. 2). However, both *casp3a* and *casp7* are transcribed in quiescent NSCs (Fig. S3A). We observe a correlation between Cas3* IHC and *Cas^CRE^Atlas* events in the number and spatial distribution in response to CPT (Fig. 6), strongly suggesting that *Cas^CRE^Atlas* reads, in part, Cas3*. Finally, when Atf3 function is abrogated, *Cas^CRE^Atlas* is still induced in NSCs, but its associated fate bias is abolished (Fig. 5K), suggesting that Atf3 is either a mediator of non-apoptotic Cas3*/Cas7* events or a parallel and converging actor. There remain, however, several missing steps in our molecular understanding, among which a key issue is the link between Cas3*/Cas7* and Atf3. The apparent non-correlation of *casp3a* and *atf3* transcripts under physiological conditions could mean that these two factors are functionally unrelated, or that they act in temporally distinct windows along the non-apoptotic process studied here. It remains that non-apoptotic Cas3*/Cas7* and Atf3 functionally interact, even if this does not prove their action along a single pathway. The relationship between non-apoptotic Cas3*/Cas7* and Atf3 is also not a simple hierarchy: overexpressed Atf3 can induce *Cas^CRE^Atlas* (although such experiments can mimic the activity of related factors) (Fig. 5F), while loss-of-function assays reveal that Atf3 is also necessary downstream or in parallel of Cas3* (Fig. 5K). Such dual positioning of Atf3 is reminiscent of previous observations; Atf3 has been described both upstream and downstream of Cas3* (Lu et al., 2007; Syed et al., 2005). Solving the relationship between Atf3 and Cas3*/Cas7* in adult NSCs will be an important next step to deepen our work, but this may require a more sensitive Cas3*/Cas7* readout, detecting Atf3 protein, or tracing Atf3^pos^ events over time. Conducting the same comparison under challenge (such as CPT, irradiation or lesion), and epistasis analyses between *Cas^Cre^Atlas* events and Atf3 expression in these contexts, could provide further information. However, with our tools fish survival proved to be technically challenging. Atf3 targets in adult NSCs also remain to be identified, as well as Cas3*/Cas7* effectors, given that blocking Atf3 does not fully abolish direct neuronal differentiation (Fig. 5K). We also do not identify the functionally relevant Cas3*/Cas7* substrates that will bias NSC fate. Intense DNA damage, which can be caused by Caspase activation (upon cleavage of the caspase-activated DNase inhibitor) and trigger cell differentiation, e.g. in muscle (Larsen et al., 2010), is here efficiently repaired in adult pallial NSCs (Fig. 7A-D_3_). Finally, *Cas^CRE^Atlas* is also induced in NSCs that follow other endogenous fates, such as symmetric NSC-NSC divisions (Fig. 3D). Thus, Cas3*/Cas7* alone is not sufficient to encode the direct differentiation fate in NSCs and a further level of regulation must exist.

Finally, we show that Cas3*/Cas7* events, and the associated fate of direct neuronal differentiation, can be engaged under non-physiological conditions of NSC stress (e.g. ionizing radiations) or neuronal repair (e.g. mechanical lesion). In the latter situation, the biased fate towards lineage termination of Cas3*/Cas7*^pos^ NSCs appears to be enhanced (Fig. 7K), which may accelerate neuron generation for repair. This occurs without apparent change in the neurogenic mode of *Cas^Cre^Atlas* events, i.e. a predominant generation of single neurons (Fig. 7L). As such, it differs from the post-lesion symmetric neurogenic divisions described by Barbosa et al. (2015).

At present, we do not know what induces Cas3*/7*/Atf3 pathways physiologically. In adult mouse muscle satellite cells, direct SC differentiation is increased upon abrogation of Notch signaling or under regeneration (Bjornson et al., 2012; Ismaeel et al., 2024; Mourikis et al., 2012). Notch3 signaling is also a major gatekeeper of NSC quiescence in the adult pallium (Alunni et al., 2013; Chapouton et al., 2010) and whether abrogating this pathway or quiescence will result in mobilizing direct neuronal differentiation fates remains to be tested. Neuronal differentiation and (neuro)epithelial delamination, as undergone by delaminating NSCs in the adult pallium, also involve major cellular remodeling (Kasioulis and Storey, 2018; Kuijpers and Hoogenraad, 2011), which may exploit Caspase (Unsain and Barker, 2015) or Atf3 (Rohini et al., 2018) activities. Finally, it will also be interesting to determine whether the neurons issued from direct neuronal conversion of NSCs have specific structural features and identity.

## MATERIALS AND METHODS

### Tools and reagents

All tools and reagents are listed in Table S1.

### Fish lines

Wild-type (AB) and *Tg(GFAP:eGFP)* (Bernardos and Raymond, 2006), *Tg(ubi:SecA5)* (van Ham et al., 2010), *Tg(her4:ERT2CreERT2)* (Boniface et al., 2009), *Cas^Cre^Atlas* (see below), *Tg(βactin:lox-stop-lox-hmg2B-mcherry)* (Wang et al., 2011) transgenic zebrafish were used. Embryos/larvae up to 5 dpf were maintained and staged as described (Kimmel et al., 1995). Adult zebrafish were maintained using standard fish-keeping protocols by the Ethics Committee n°39 of Institut Pasteur (authorization #36936) and DDPP-2021-921 of the Direction Départementale de la Protection des Populations de Paris.

### Plasmid/vector construction and transgenesis

The *mCD8-DEVD-V5-Cre* (*CDVC*) construct was PCR-generated by fusing in-frame the *mCD8-Diap1* region of a plasmid encoding *Drosophila Casexpress* DQVD (Ding et al., 2016) to a *V5-CRE* recombinase cassette and subcloned into the Tol2-kit vector *pME-MCS* to generate *pME-CDVC*. *pME-CDVC* was used to generate the transgenesis vector *pTol2-HCDVC* (*her4:mCD8-diap1-V5-CRE-SV40pA*) using the L/R recombinase reaction and the Tol2 vectors p302 and p395, and the 5′ vector *N11*. In the final product, the DEVD caspase site was mutagenized to GSGC to generate the control plasmid pTol2-HCDVC* by the Round-the-horn mutagenesis method (https://openwetware.org/wiki/%27Round-the-horn_site-directed_mutagenesis). Transgenic lines were made by injecting one-cell embryos with a mix containing 60 ng/µl of plasmid and 60 ng/µl of *transposase* capped RNA.

The *atf3* cDNAs was amplified from reverse-transcribed 16 hours post-fertilization (hpf) embryo RNA and subcloned into *pSCA*. The *atf3* RNA probe was generated from the long version of the RNA (full coding sequence). The *atf3-P2A-GFP* constructs were generated with the Gibson method and subcloned into the *pCMV5* vector using the NEBuilder^®^ HiFi DNA Assembly Cloning Kit.

### RT-PCR for the validation of efficiency of *atf3* vivoMO

cDNA, extracted from 24 hpf embryos, was amplified by RT-PCR using primers *atf3_FL_fwd* and *atf3_FL_rev* and the following cycles: 98°C, 1 min; 98°C, 30 s; then 35 cycles of 98°C, 10 s; 63°C, 30 s; 72°C, 20 s; then 72°C, 2 min.

### IHC

Brains were dissected in 1× PBS at 4°C, their tela choroida was manually removed and the brains were directly transferred to a 4% paraformaldehyde solution in PBS for fixation. They were fixed overnight at 4°C under permanent agitation. After four washing steps in PBS, brains were dehydrated through a 5-10 min series of 25%, 50% and 75% methanol diluted in 0.1% Tween-20 PBS solution and kept in 100% methanol at −20°C. Rehydration was performed using the same solutions, and then brains were processed for whole-mount IHC. After rehydration, the telencephala were dissected out and subjected to an antigen retrieval step using HistoVT One for 1 h at 65°C. Brains were rinsed three times for at least 10 min in a 0.1% DMSO and 0.1% Triton X-100 PBS 1× solution (PBT) and then blocked with 4% normal goat serum in PBT (blocking buffer) 4 h at room temperature on an agitator. The blocking buffer was later replaced by the primary antibody solution (diluted in blocking buffer), and the brains were kept overnight at 4°C on a rocking platform. The next day, brains were rinsed five to ten times over 24 h at room temperature with PBT and incubated in a solution of secondary antibodies diluted in PBT overnight, in the dark, and at 4°C on a rocking platform. In some instances, to be able to use the ZO1 dye-coupled antibody in the presence of another primary mouse mAB, secondary antibody free sites were blocked by incubation with 2% mouse serum in PBT for 1 h before applying the dye-coupled ZO1 antibody. After three rinses in PBT over 4 h, brains were transferred into PBS. Dissected telencephala were mounted in PBS on slides using 0.5 mm-thick holders. The slides were sealed using a glue gun.

Primary antibodies were used at a final concentration of 1:1000 for chicken anti-GFP and chicken anti BrdU, 1:500 for dye-coupled-ZO1, 1:300 for Casp3a, 1:500 for RFP, 1:200 for Sox2, ZO1 and mAb anti-GFP, and 1:100 for γH2AX. Secondary antibodies were all used at a final concentration of 1:1000.

### Whole-mount ISH and IHC

ISH was performed as described previously (Bosco et al., 2013; Chapouton et al., 2010; Ninkovic et al., 2005) except for the additional presence of 5% dextran sulfate during the hybridization phase. For combined ISH and IHC, the ISH was developed using Fast Red. See Table S1 for details of antibodies and probes used in this study.

### Ventricular micro-injections and electroporation

Micro-injections into the adult brain ventricle were performed on anaesthetized fish as described (Rothenaigner et al., 2011) except that DNA was injected at the midbrain midline to avoid damaging the pallium. vivoMOs (Gene Tools) were injected at a concentration of 0.125 mM. Micro-injections into 4 dpf larval hindbrain ventricle were performed on anesthetized larvae immobilized in 4% methyl cellulose.

For electroporation, plasmid DNA was diluted to 1 µg/µl in 0.1×PBS and injected into the ventricle. Electrodes (Tweezertrodes, 5 mm platinum) were placed on each side of the fish head. Fish were then administered two electric pulses (70 V, 50 ms width, 1000 ms space).

### Drug treatments

#### 4-OHT treatments and BrdU incorporation

4-OHT treatment was performed as previously described (Mosimann et al., 2011) on *her4:Ert2CreErt2*, *ßactin:LoxSTOPloxhmgbmCherry*. Clonal recombination conditions at 1 mpf were 10 min with 0.5 µM 4-OHT as described by Than-Trong et al. (2020. They were followed by a 4 h pulse of 1 mM BrdU. *Cas^Cre^Atlas* were only treated with 1 mM BrdU for 4 h. Fish were then washed four times, transferred into fresh fish water, and grown as usual until 2 months of age.

#### CPT treatment

CPT was dissolved as a 10 mM stock in DMSO, aliquoted and stored frozen until use. Just before use, an intermediate solution was prepared in DMSO and further diluted 50 times in PBS (for injections) or fish water (for incubations) to reach the working concentration.

### X-ray irradiation

Fish (3-5 months old) were placed into 2-l cages inside an X-ray irradiator (Gulmay CP160/10, 250 kV, 12 mA) and irradiated for the length of time needed to reach the expected dose (5 Gy= 212 s). Controls were placed for the same duration in the chamber but left untreated. Following irradiation, fish were kept in 6-l cages in a 28°C incubator for the desired length of time. Fish were fed once a day and water changed daily if needed. Fish brains were then dissected, their tela choroida was removed, and the brains were then fixed for ISH or IHC.

### Imaging and image analysis

Images of whole-mount immunostained telencephali were acquired on a confocal microscope (LSM700 and LSM710, Zeiss) using a 20× objective or a 40× oil objective (Plan-Apochromat 40×/1.3 Oil M27) and tile images of four to eight *z*-stacks were stitched with ZEN2009 software. 3D renderings were generated using Imaris® software (versions 8 and 9, Bitplane). Vertical plane images were extracted when needed. *Cas^CRE^Atlas* clones were resolved manually using 3D rendering and the slice mode and highlighted in different colors. For Fig. 7A-D and Fig. S3, a nuclear mask was created in Imaris using Sox2 nuclear staining in order to quantify nuclear H2AX staining. For Fig. 4D, single planes at different depth were extracted.

For dorsal whole-mount views of the telencephalon (ISH in blue), images were taken using a Nikon macroZoom.

### Statistics

All experimental data were analyzed using Prism software and are expressed as mean±s.e.m. Significance was set at *P*<0.05. Global comparison of proportions between experimental and control conditions (Figs 3D, 5D-E, 6H, 7K,L, Figs S2B, S4C) were performed via a contingency test based on a χ^2^ analysis. When a statistically significant difference was detected, the specific cell fate category(ies) significantly different between experimental and control datasets was determined using a Fisher's exact test with Bonferroni correction to account for multiple comparisons (corresponding to the number of categories tested). The number of fish tested is indicated in the legend for each figure; we report the number of hemispheres tested, and always only analyzed one hemisphere per fish. All counts are provided in Table S2.

## Supplementary Material



10.1242/develop.204381_sup1 Supplementary information

Table S1. Tools and reagents

Table S2. Raw counts for the different experiments
