# Supplementary material for: Non-apoptotic caspase events and Atf3 expression underlie direct neuronal differentiation of adult neural stem cells
Source: Development. 2024 Nov 20;151(22):dev204381. doi: 10.1242/dev.204381 (PMC11607687; doi:10.1242/dev.204381)
Supplement: Supplementary information [file develop-151-204381-s1.pdf]

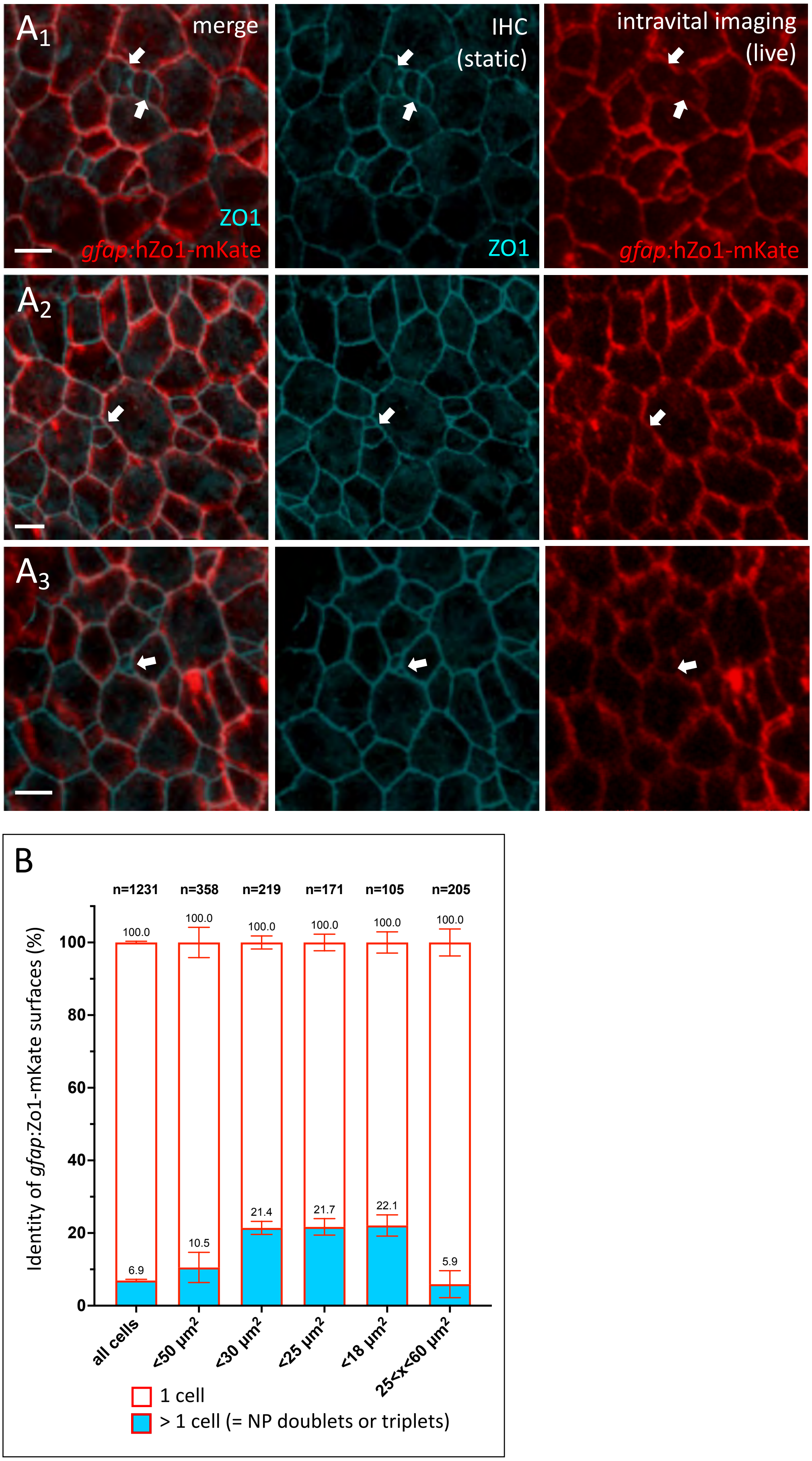

Development • Supplementary information

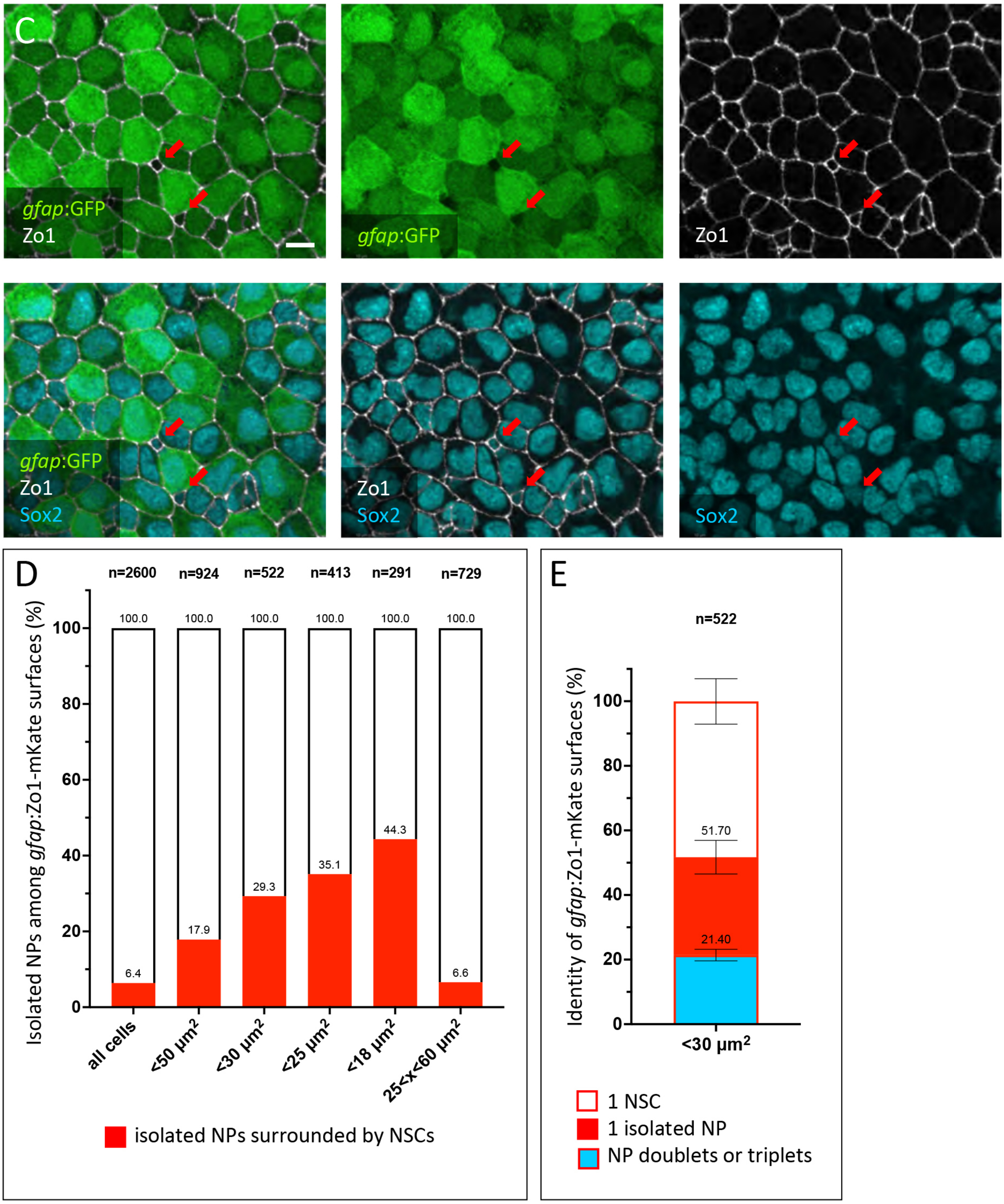
C
gfap:GFP
Zo1
gfap:GFP
Zo1
gfap:GFP
Zo1
Sox2
Zo1
Sox2
Sox2
D
n=2600
n=924
n=522
n=413
n=291
n=729
Isolated NPs among gfap:Zo1-mKate surfaces (%)
100.0
6.4
17.9
29.3
35.1
44.3
6.6
all cells
<50 µm2
<30 µm2
<25 µm2
<18 µm2
25<x<60 µm2
isolated NPs surrounded by NSCs
E
n=522
Identity of gfap:Zo1-mKate surfaces (%)
51.70
21.40
<30 µm2
1 NSC
1 isolated NP
NP doublets or triplets

Fig. S1. Estimation of the proportion of Zo1-mKate apical surfaces corresponding to NPs in the intravital imaging approach. A_1-A_3. Three examples of whole-mount overlays between Zo1-mKate fluorescence at a final imaging tp (red) and Zo1 immunohistochemistry (IHC) on the same brains following fixation (cyan) (Da pallial domain). The merge panel shows the unambiguous alignment of the fixed and live images, except for some adjacent NPs (white arrows), which cannot be recognized as distinct apical domains in the live image because expression of the Zo1-mKate fusion protein is driven by the NSC *gfap* regulatory elements. Scale bars: 10 µm. **B.** Proportion of Zo1-mKate surfaces corresponding to NP clusters (cyan) versus single cells (white), estimated when comparing Zo1-mKate (live) and Zo1 (fixed). The percentage is estimated for different cut-offs of apical surface areas (in μm^2). Da pallial domain, n=2 brains. **C.** Two examples of isolated NPs, surrounded by NSCs. Whole-mount views of the pallial NSC/NP population in a Tg(*gfap:GFP*) adult, where triple IHC reveals NSCs (*gfap*:GFP^{pos}, green), Zo1 (white) and all progenitors (Sox2, blue). Isolated NPs are indicated by red arrows. **D.** Proportion of Zo1 surfaces corresponding to NPs isolated among NSCs, quantified on Tg(*gfap:gfp*) pallia following Zo1 IHC. The percentage is estimated for different cut-offs of apical surface areas (in μm^2). Dm pallial domain, n=2 brains. **E.** Compiled from B and C, identity of Zo1-mKate surfaces < 30 μm^2. 48.3% are NSCs (white), 51.7% are NPs (isolated NPs -red- or NP groups -cyan-).

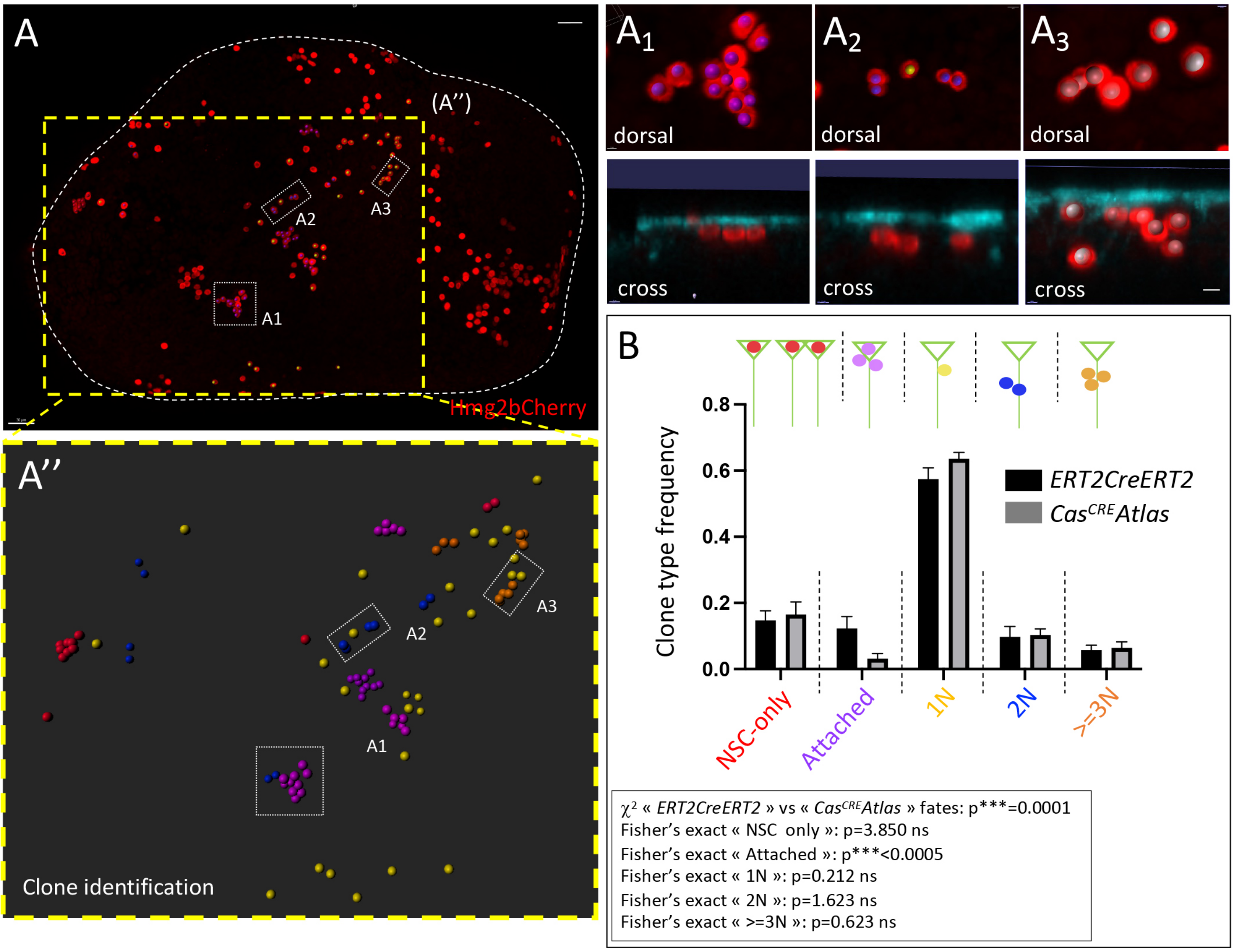

Fig. S2. Clonal analysis of *Cas^Cre^Atlas*-driven fates with details on "neuron-only" clones. A-A''': Segmentation of clones from Fig.3C. A: Hmg2bmCherry channel only. A_1-A_3: examples of several clones (indicated on A), with color-coded segmentation, views from dorsal (top) and in optical cross sections (bottom) -not all cells are visible-. Clones in A_1: "attached" (magenta); clones in A_2: "1N" (single neuron) (1 clone, yellow) and "2N" (neuron doublet) (2 clones, blue); clones in A_3: "1N" (single neuron) (2 clones, yellow) and ">=3N" (3 neurons or more, neurons only) (1 clone, orange); A'': Imaris segmentation of unambiguously identifiable clones in the area shown in A (yellow dotted square). **B.** Frequency of the different Hmg2bmCherry-positive clone types (represented schematically at the top; green triangles:

Development • Supplementary information

NSCs; colored dots: Hmg2bmCherry-positive cells) generated from *her4*-positive NSCs between 1 and 2 mpf in the *her4:ERT2CreERT2* and *Cas*^{CRE}*Atlas* backgrounds (black and gray bars, respectively) (same data as in Fig.3D with subdivision of neuron-only clones), graph displaying results with SEM. Statistical analysis for global differences between *ERT2CreERT2* and *Cas*^{Cre}*Atlas* fates: contingency Chi-square test: p***=0.0001. Statical analyses for individual fates among 5 possible fates (NSC-only, Attached, 1N, 2N and >=3N: Fisher's exact test with Bonferronni correction: NSC-only p=3.850 ns, Attached: p***<0.0005, 1N: p=0.212 ns, 2N: p=1.623 ns, >=3N: P=0.623 ns. Scale bars: A, A_1-A_3: 30 µm.

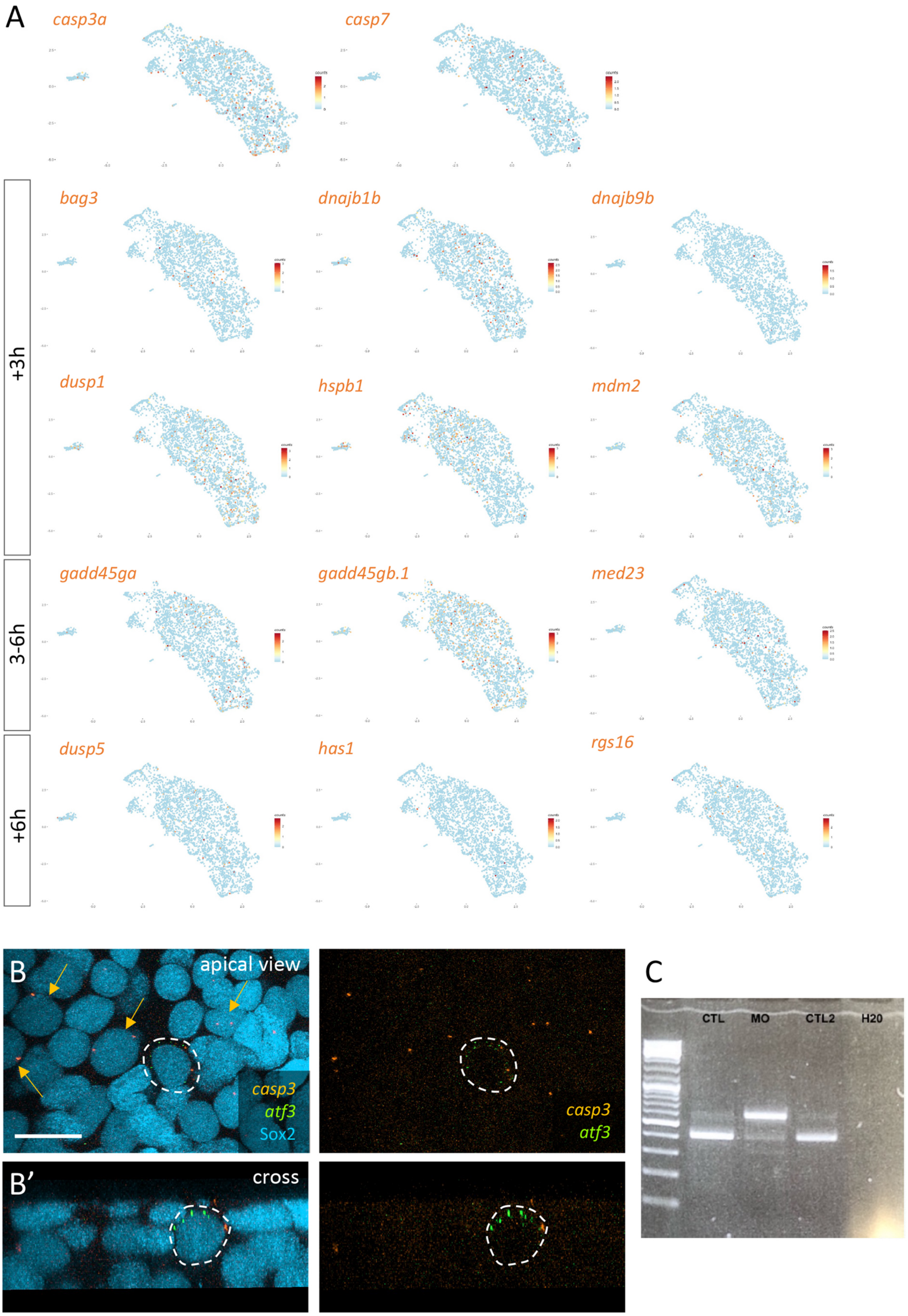

Development • Supplementary information

Fig. S3. Expression of apoptosis/anastasis mediators in zebrafish adult pallial NSCs and validation of the *atf3* MO. A. Cells expressing the genes indicated (orange dots, UMI values color-coded) positioned on the scRNAseq UMAP of adult quiescent NSCs (gray dots). The candidate genes are taken from (Sun et al., 2017; Tang et al., 2017). The positions of NSCs closest to activation and/or neurogenesis commitment and NSCs in a deep/long quiescence phase are as in Fig.2A. **B.** Analysis of *atf3* and *casp3a* cotranscription using whole-mount ISH with RNAscope Hiplex probes (*atf3*: green, *casp3a*: orange), revealed together with Sox2 (cyan, IHC); apical (top) and cross (bottom) views are shown. An *atf3*^{pos},*casp3a*^{pos} cell is circled, and orange arrows point to other *casp3a*^{pos} cells that are *atf3*^{neg}. Scale bar: 10µm. Among 1025 cells counted in a pallial region of interest, 54 expressed *atf3* and 147 (15%) expressed *casp3a*. Among *atf3*^{pos} cells, 21% express *casp3a*. The proportion of *casp3a*^{pos} cells overall is therefore of the same order as within the *atf3*^{pos} population, arguing against a specific enrichment. **C.** RT-PCR for *atf3* in 24hpf embryos treated as follows: CTL: non-injected, MO: injected at the one-cell stage with 125mM of *atf3* vivoMO, CTL2: injected at the one-cell stage with 125mM of control vivoMO, H20: no RNA.

Development • Supplementary information

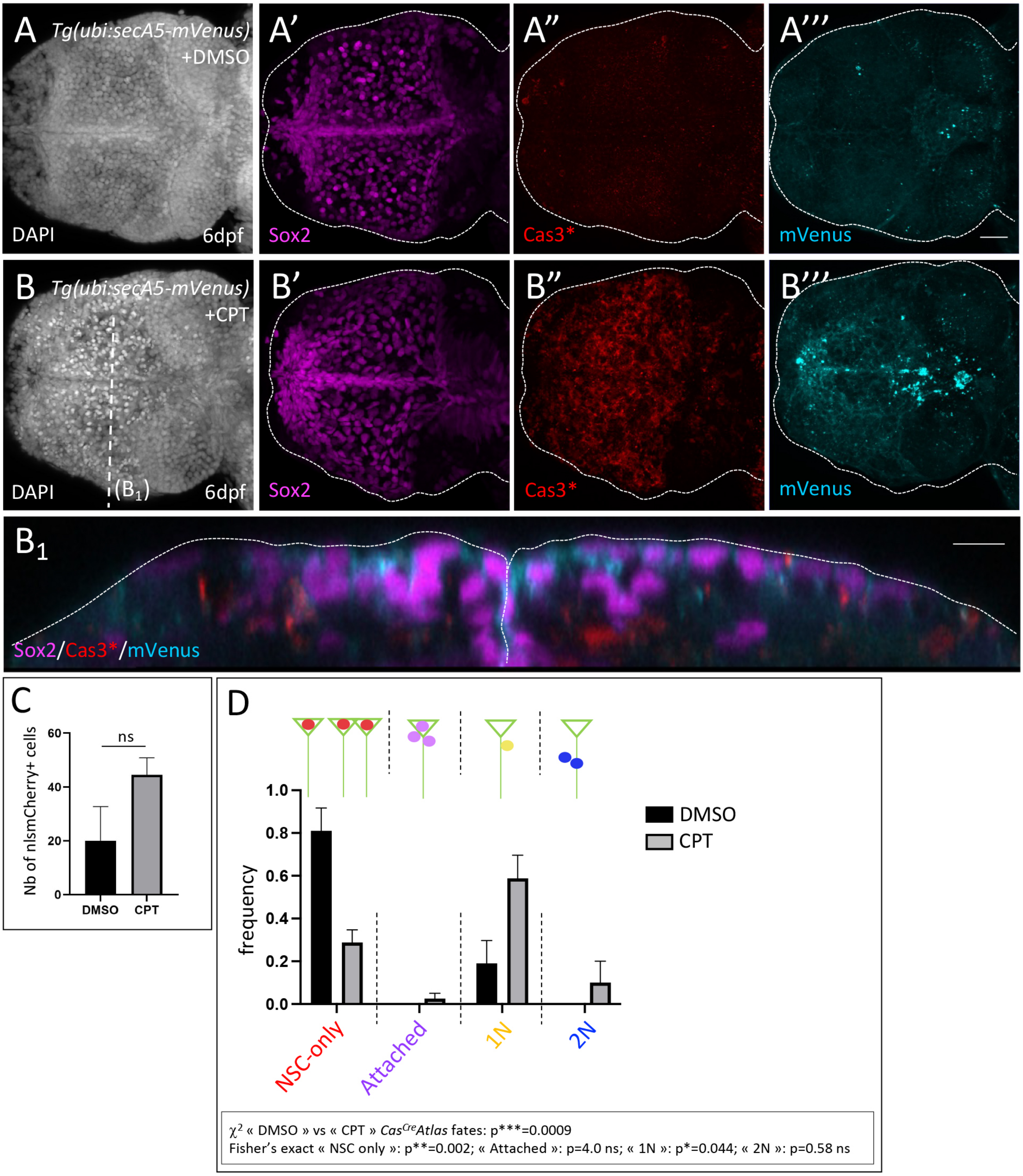
A
Tg(ubi:secA5-mVenus)
+DMSO
DAPI
6dpf
A'
Sox2
A''
Cas3*
A'''
mVenus
B
Tg(ubi:secA5-mVenus)
+CPT
DAPI
(B1)
6dpf
B'
Sox2
B''
Cas3*
B'''
mVenus
B1
Sox2/Cas3*/mVenus
C
Nb of nlsmCherry+ cells
ns
DMSO
CPT
D
frequency
DMSO
CPT
NSC-only
Attached
1N
2N
χ2 « DMSO » vs « CPT » CasCreAtlas fates: p***=0.0009
Fisher's exact « NSC only »: p**=0.002; « Attached »: p=4.0 ns; « 1N »: p*=0.044; « 2N »: p=0.58 ns

Fig. S4. Experimentally induced Cas3* events drive NSC death in the larval pallium and direct neurogenesis from NSCs in the adult pallium. A,B. Effect in larvae. Tg(*ubi:secA5-mVenus*) 6dpf larvae were incubated overnight in DMSO (A) or CPT (B) (400nM). The brains were dissected and subjected to whole-mount IHC for Sox2 (magenta), Cas3* (red) and mVenus (cyan). B_1 is an optical cross-section of B at the level indicated. Note that the mVenus staining is ventricular and affects Sox-positive NSC/NPs. Scale bars: A-A3''',B-B''': 15µm, B1: 10µm. **C,D.** *Cas^{Cre}Atlas* fates induced in the adult pallium upon CPT treatment. Details from dataset presented in Fig.6D-. (C) Quantification of Hmg2bmCherry-positive cells located within 2-3 cell rows from the ventricle at 7 days post-treatment. Graph displays results with SEM. Statistical analysis: Welsh's t test: not significant (p=0.17). (D) Details on neurons. Same dataset as in Fig. 6H but with clones corresponding to neuron singlets (1N) and doublets (2N) analyzed separately. Graph displaying results with SEM. Statistical analysis for global differences between DMSO and CPT fates: contingency Chi-square test: p***=0.0009. Statical analyses for individual fates among 4 possible fates (NSC-only, Attached, 1N and 2N: Fisher's exact test with Bonferronni correction: NSC-only p**=0.002, Attached: p=4.0 ns, 1N: p*=0.044, 2N: p=0.58 ns.

Development • Supplementary information

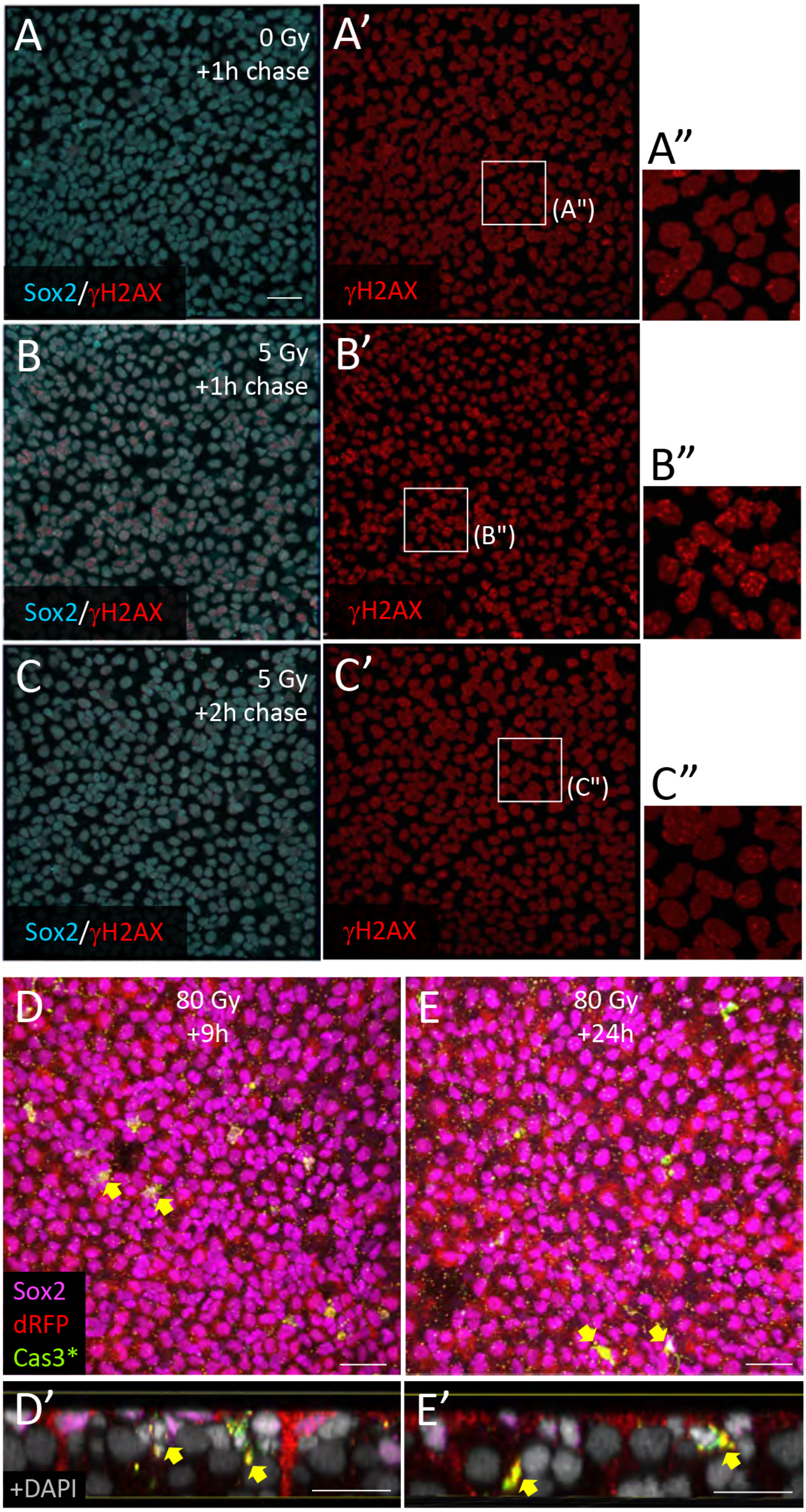

Development • Supplementary information

Fig. S5. Time course of the effect of X-rays on NSCs *in vivo*. A-C″. DNA repair response to irradiation in NSCs. γH2AX DNA repair foci (red) in NSCs/NPs (Sox2+, cyan) revealed by whole-mount IHC on adult pallia under control (A,A′) or irradiation conditions (B-C′) (5 Gy for 1hr followed by 1 or 2hr chase). γH2AX DNA repair foci are prominent 1h after treatment but resolved after 2h. Scale bar: 10 μm. **D-E′.** Time course of Cas3* induction at high X-ray doses. Whole-mount pallia from Tg(her4:dRFP) adult fish processed for immunohistochemistry for Sox2, dRFP and Cas3* (color-coded) after irradiation was applied at 80 Gy for 9h (D) or 24h (E). Yellow arrows in D, E point to Cas3*-positive cells that are also visible in the optical cross sections respectively in D′, E′ (which also display the DAPI counterstaining). Scale bar: 10 μm.

Table S1. Tools and reagents

Available for download at
https://journals.biologists.com/dev/article-lookup/doi/10.1242/dev.204381#supplementary-data

Table S2. Raw counts for the different experiments

Available for download at
https://journals.biologists.com/dev/article-lookup/doi/10.1242/dev.204381#supplementary-data
