# Supplementary material for: Non-apoptotic caspase events and Atf3 expression underlie direct neuronal differentiation of adult neural stem cells
Source: Development. 2024 Nov 20;151(22):dev204381. doi: 10.1242/dev.204381 (PMC11607687; doi:10.1242/dev.204381)
Supplement: Supplementary file 1 [file develop-151-204381_review_history.pdf]

# Non-apoptotic caspase events and Atf3 expression underlie direct neuronal differentiation of adult neural stem cells

Frédéric Rosa, Nicolas Dray, Sébastien Bedu and Laure Bally-Cuif
DOI: 10.1242/dev.204381

**Editor**: Steve Wilson

**Review timeline**

| | |
|---|---|
| Original submission: | 12 March 2024 |
| Editorial decision: | 22 April 2024 |
| First revision received: | 9 September 2024 |
| Editorial decision: | 30 September 2024 |
| Second revision received: | 4 October 2024 |
| Accepted: | 8 October 2024 |


---

**Original submission**

First decision letter

MS ID#: dev.204381

MS TITLE: Non-apoptotic caspase events and Atf3 expression underlie direct neuronal differentiation of adult neural stem cells

AUTHORS: Frédéric Rosa, Nicolas Dray and Laure Bally-Cuif

I have now received all the referees' reports on the above manuscript, and have reached a decision. The referees' comments are appended below, or you can access them online: please go to BenchPress and click on the 'Manuscripts with Decisions' queue in the Author Area.

As you will see, the referees express considerable interest in your work, but have some criticisms and suggestions for improving your manuscript - both in terms of additional experiments and the way that some aspects of the study are presented. If you are able to revise the manuscript along the lines suggested, I will be happy receive a revised version of the manuscript. If it would be helpful, you are welcome to contact us to discuss your revision in greater detail. Please send us a point-by-point response indicating your plans for addressing the referees' comments, and we will look over this and provide further guidance.

Please attend to all of the reviewers' comments and ensure that you clearly highlight all changes made in the revised manuscript. Please avoid using 'Tracked changes' in Word files as these are lost in PDF conversion. I should be grateful if you would also provide a point-by-point response detailing how you have dealt with the points raised by the reviewers in the 'Response to Reviewers' box. If you do not agree with any of their criticisms or suggestions please explain clearly why this is so.

Reviewer 1

*Advance summary and potential significance to field*

In their submitted manuscript Rosa and co-workers present a compelling case for a hitherto unrecognized role of anastasis in the neurogenesis of the zebrafish brain. In the manuscript they

© 2024. Published by The Company of Biologists under the terms of the Creative Commons Attribution License (https://creativecommons.org/licenses/by/4.0/).

reanalyse earlier datasets from the Bally-Cuif lab and also develop an innovative new genetic tool, the CasCRE Atlas to correlate delamination events observed in the ventricular, neurogenic zone of the zebrafish pallium, with the occasional activation of Caspase3 in the same cells.
Overall I think that the mechanism presented in the paper will be of great interest for the readers of Development. The correlation between delamination events, Cas3 activation and the presence of single neuron clones is convincing. On the other hand, the suggested link between Cas3 activation and atf3 expression needs further confirmation in my opinion, and the authors should also keep an open mind about an inverse causality (i.e. under homeostatic conditions Atf3 expression could drive Cas3 activation), see my comments below .

Comments for the Author

Major points and remarks:
- A major point in the mechanistic explanation provided by the authors hinges on the purported induction of atf3 by Cas3*. Yet, in the manuscript there is no evidence presented for Cas3* - atf3 mRNA, or CasCREAtlas -atf3 mRNA colabelling. If the maturation of Hmg2bmCherry is slow, as the authors seem to suggest, could an HCR for mCherry (which would amplify the signal), or a double cas3 - atf3 fluorescent in situ help in providing this crucial piece of evidence?
- The timing between atf3 expression and Cas3 activation is especially intriguing as the literature has plenty of papers which suggest that Atf3 itself is necessary for the cleavage of Cas3, as the authors themselves acknowledge at one point. (The fact that no significant change can be observed in delamination events in atf3-L-P2A-nlsGFP or atf3-vivoMO injected animals, seems to suggest an upstream role for the activation of the caspase pathway, but due to the low frequency of such events, such an inference should be treated with due caution.)
- Is there a reason why clone type sizes are not reported for the irradiation experiments (Figure 7A-D3), like for all the others?
- In animals irradiated with 40 Gy the authors assay cell death 24 hours post irradiation (hpi). Yet, in other experiments - albeit performed in larval animals - after a 25 Gy irradiation a peak in apoptosis can be seen 3.5 hpi (PMID: 26753623). Isn't 24 hpi way too late to observe the effects of the irradiation? Perhaps most Caspase-3 activation due to the irradiation was resolved by this time either by cell death, or by anastasis.
- At the very end of the Results section (lines 331-332) the authors declare that: "At 7dpl, CasCREAtlas fates recorded in lesioned hemispheres did not detectably differ". While the difference is not significant, a very strong trend seems to be present in the reduction of NSCs and increase of single neuron clones in the affected hemipallia of lesioned animals. In this case I think a compelling case can be made for an increase in sample size (especially as 11 hemipallia were used for the controls, but only 3 for the lesioned animals), as it likely could lead to a significant result.

Minor remarks:
- Single neuron clones should be labelled in yellow, for consistency - see Figure 6H and line 300 in the text.
- Expressions with Latin origin, such as in situ and in vivo should be always italicized.
- For consistency, “Ventricular" should start with a capital letter in Figures 5D,E.
- In Figure S4C radiation strength and chase time are missing.
- The reference for the Morizet et al. preprint is incomplete.

Reviewer 2
Advance Summary and Potential Significance to Field:
This work identifies molecular mechanisms underlying the direct conversion of neural stem cells (NSCs) in the adult zebrafish brain, by focusing on the potential role of non-apoptotic caspase activation in cell fate. To do so, the authors explore caspase activation events that do not result in apoptosis and the role of the transcription factor atf3, which is a known mediator of non-apoptotic caspase activation events in other species. The work, therefore, addresses a poorly understood and interesting process by proposing that processes of caspase activation not resulting in apoptosis may have an effect in cell fate decisions.

*Comments for the author*

© 2024. Published by The Company of Biologists under the terms of the Creative Commons Attribution License (https://creativecommons.org/licenses/by/4.0/). 

This work identifies molecular mechanisms underlying the direct conversion of neural stem cells (NSCs) in the adult zebrafish brain, by focusing on the potential role of non-apoptotic caspase activation in cell fate. To do so, the authors explore caspase activation events that do not result in apoptosis and the role of the transcription factor atf3, which is a known mediator of non-apoptotic caspase activation events in other species. The work, therefore, addresses a poorly understood and interesting process by proposing that processes of caspase activation not resulting in apoptosis may have an effect in cell fate decisions. The authors have developed, over the last few years, very innovative and rigorous in vivo imaging strategies by using reporters and live microscopy, followed by quantitative analysis, to analyze adult NSC behavior in the fish pallium at the single cell level, positioning their studies at the forefront of those devoted to cell fate decisions in adult neurogenesis.
Previous studies by the same group have demonstrated the process of NSC direct conversion, meaning the direct differentiation of NSCs into neurons without previous cell division. This has not been conclusively proven in mammals, despite some data in the dentate gyrus, but many similarities between zebrafish and rodent adult NSCs make these studies relevant and offer new hypotheses to be tested in the rodent brain.

The authors first rigorously test the strains of transgenic fish that are required to address the hypothesis. To do so, they characterize NSC direct conversion in a strain expressing a marker of apical surfaces (ZO1-mKate2) and a marker of neurogenic fate (delta) and cleverly use experimental timelines and cell dimensions to focus on the cell dynamics of NSCs vs. other neural progenitor (NP) cells in direct conversion to a neuronal fate without cell division. To detect caspase activation they make use of a fish strain (Cas-cre Atlas) that expresses, under the control of an NSC promoter (her4), a cre recombinase targeted to the cell membrane and whose release from the plasma membrane is dependent on a caspase target peptide (DEVD). This reporter line, which is sensitive to caspase activation is also thoroughly tested in the manuscript. A control line expressing a target peptide for other proteases is used as a control. The authors elegantly use lineage tracing (from the Cas-cre Atlas line and a transgenic line for conditional cre induction in NSCs) and BrdU pulse-chase experiments combined with their experience in imaging direct conversions and describe that caspase-mediated effects correlate with a bias of NSC fate towards direct neuronal differentiation. Although a causal relationship cannot not be demonstrated, because the caspase activation is transient but the recombination is stable, and furthermore some activations may lead to cell death, the association is sufficiently strong. Furthermore, induction of caspase activation by apoptotic inducer camptothecin results in direct conversion.

With the proposal that the observations could be the result of the cleavage of signaling pathways effectors by caspases, they focus the second part of their work on atf3. The authors show that only a few delaminating NSCs in the adult pallium express a long form of atf3 mRNA. Loss- and gain-of-function experiments demonstrate that atf3 regulates direct conversion. The authors then analyze direct conversion in conditions of apoptosis induction by irradiation and by mechanical lesion to the pallium. Although the former does not induce the process, despite lesions in the DNA, the latter results in more atf3-positive NSCs and delaminating cells.

The work is relevant and well executed. Some recommendations for improvement are proposed.

Regarding experiments, the authors should better connect caspase activation with atf3. One suggestion would be to use camptothecin in Cas-cre Atlas adults injected with the morpholino (MO) against atf3-L. This is especially relevant as they show in Figure 7 that a mechanical injury increases the numbers of cells expressing atf3-L but this could be a result of the lesion independent of the caspase activation.

Why are the experiments of overexpression of atf3 and MO shown in Figure 5 performed differently? They need to homogenize these data.

The results in experiments shown in Figure 7E-K are not clearly stated. As explained in the text, it appears that more delaminating cells expressing atf3 are observed in the lesioned hemisphere at 3 days post lesion, but this is not quantitated. The quantitation is shown at 7 days post lesion but no significant differences are found (Figure 7K). That together with a rather unclear description in the text does not clarify whether the mechanical lesion truly induces direct conversion for repair. If this is so, the authors could try to see if atf3 MOs interfere with the repair response.

© 2024. Published by The Company of Biologists under the terms of the Creative Commons Attribution License (https://creativecommons.org/licenses/by/4.0/).

The authors need to improve the way they explain the experiments, especially for those who do not work in zebrafish. For example, they need to include a clear timeline for the development of zebrafish and how the different experiments relate to this timeline. That could be done in coordination with the schemes used in the different figures (for example, Figure 2 and 3). In addition, they should always use the same timings (for example, mpf and wpf are used in different parts of the manuscript).

Also, please try to homogenize the graphs (Figures 3D, 5D, K, and 7K).
Furthermore, in these graphs, the attached and detached categories include the other ones (Single Neu, Neu doublet, NSC clones Single NSCs) and therefore should be represented in different graphs for clarity. Also, try to use the same order and categories throughout the graphs, especially as they need to be compared.

The authors need to name the atf3 forms (short and long) with their nomenclature before they use them in the electroporation experiments so the latter can be more easily followed.

Minor things:
-In Figure 1A: add the symbols to the figure instead of just in the figure legend. Also, it would be clearer if all the cells, delaminating or not, would be entirely delineated.
-In Figure 3, I would suggest using the same representation of the experimental design as in Figure 2 to ease reading.
-In Figure 3A and related text, better define “induced” and “scored” clones.
-Some sentences that are difficult to understand are found in lines # 182, 270, 320, and 331.
-In line 246 an “and” between “fluorescent” and “chromogenic” is missing.
-Also in line 246, the ISH is used to address not only the “morphology”, but also the “location” of atf3-positive cells.

Reviewer 3

*Advance summary and potential significance to field*
This study explores the role of a novel pathway on the process of neuronal delamination and direct neurogenesis in the adult zebrafish brain. Specifically, the authors investigate the potential involvement of the caspase non-apoptotic pathway in neuronal delamination and direct, offering a new perspective on this phenomenon. Using Caspase reporter lines, the study aims to establish a correlation between caspase non-apoptotic activation and the occurrences of direct neurogenesis. The findings reveal an increase in single neuron clones upon Caspase activation, indicative of an increase in neuronal delamination. this study also examines the effects of caspase non-apoptotic activation and neuronal displacements following injury, although the overall outcome of this approach is written is a convoluted way. In addition this study explores the role of atf3, a mediator of non-apoptotic events, on direct neurogenesis through overexpression and knockdown (KD) experiments. The data suggest that Atf3 KD diminishes neuronal delamination, whereas overexpression amplifies these events.
Overall, this study stands out for its innovative approach to manipulating adult neurogenesis, and exploring a novel pathway on the process- a task typically full of challenges.

*Comments for the author*

The flow of the manuscript could be improved; the sequence of experiments better justified. Consistent use of nomenclature, such as NSC or NPs, throughout the manuscript would also contribute to clarity. The link between the initial morphometric analysis and subsequent sections of the study would also help improved the flow of information.

Text related to Fig1 and FigS1 should be simplified and consistent to help the reader's understanding. Gfap:ZO-1-mKate labels the apical surface of NSCs but not NPs. However, this distinction is not always clear in the text and FigS1, leading to confusion regarding the identification of NPs solely by ZO1-mKate expression.

© 2024. Published by The Company of Biologists under the terms of the Creative Commons Attribution License (https://creativecommons.org/licenses/by/4.0/). 

To improve clarity, consistent labelling and clear definitions would be helpful.
For instance, the apical surface of NPs is defined by Zo1 immunofluorescence (+) Zo1-mKate (-) expression, while that of NSCs can be identified by Zo1 immunofluorescence (+) Zo1-mKate (+) or gfap:tomato+ and Zo1 immunofluorescence (+). It would also be beneficial to provide an example of an isolated NP for clarification in FigS1.

A more detailed explanation of statistical treatment would be helpful and the number of fish per experiment should be provided. I would advise to ask a statistician to discuss the choice of statistical tests. Chi-square test tend to indicate significant differences between control and treated groups and demonstrate that observed differences are not solely due to data variability, they do not allow for the interpretation of whether the proportion of lineage type 1 is significantly increased or decreased in control compared to a treated condition.
Therefore, it is confusing that most graphics appear to compare averages between control and treated conditions as if an ANOVA was applied. However the figure legends mention Chi-square. Authors should try to improve this important part of the manuscript as this would help draw the appropriate conclusions.

Minor corrections and suggestions include clarifying whether the gfap promoter drives expression in NSCs and if her4 drives expression in NPs and NSCs.
Inconsistencies in the text regarding her4 association with both NSCs and NPs should be addressed. Additionally, on page 5, Fig.S1B should be corrected to S1C and Fig.S1C to S1D. In FigS1B, I think "1 cell" should be replaced with "1 NSC" as in FigS1D, and ">1 cell (doublets or triplets)" should be replaced with "NPs."
Consider removing "N. Dray unpub." and briefly discussing Baek et al., 2018.
Define "Tp" in the main text. Rectify misaligned diagrams in some of the figures.

---

**First revision**

Author response to reviewers' comments

**Reviewer 1**
**Advance Summary and Potential Significance to Field:**
In their submitted manuscript Rosa and co-workers present a compelling case for a hitherto unrecognized role of anastasis in the neurogenesis of the zebrafish brain. In the manuscript they reanalyse earlier datasets from the Bally-Cuif lab and also develop an innovative new genetic tool, the CasCRE Atlas to correlate delamination events observed in the ventricular, neurogenic zone of the zebrafish pallium, with the occasional activation of Caspase3 in the same cells.
Overall I think that the mechanism presented in the paper will be of great interest for the readers of Development. The correlation between delamination events, Cas3 activation and the presence of single neuron clones is convincing. On the other hand, the suggested link between Cas3 activation and atf3 expression needs further confirmation in my opinion, and the authors should also keep an open mind about an inverse causality (i.e. under homeostatic conditions Atf3 expression could drive Cas3 activation), see my comments below.

**Comments for the Author:**

**Major points and remarks:**
1- A major point in the mechanistic explanation provided by the authors hinges on the purported induction of atf3 by Cas3*. Yet, in the manuscript there is no evidence presented for Cas3* - atf3 mRNA, or CasCREAtlas - atf3 mRNA colabelling. If the maturation of Hmg2bmCherry is slow, as the authors seem to suggest, could an HCR for mCherry (which would amplify the signal), or a double cas3 - atf3 fluorescent in situ help in providing this crucial piece of evidence?

We now attempted to test co-expression in three ways:

- *atf3* ISH and *mCherry* ISH in the Cas$^{Cre}$ Atlas background

© 2024. Published by The Company of Biologists under the terms of the Creative Commons Attribution License (https://creativecommons.org/licenses/by/4.0/).

- *atf3* ISH and *casp3* ISH in wildtype pallia
- *atf3* ISH and an immunohistochemistry for activated Cas3* in wildtype pallia

We used RNAscope probes to work with highest sensitivity. For unknown reasons, we failed to detect *mCherry* transcripts (we had some issues with some probes from BioTechne, and did not have time to troubleshoot). But we analyzed the two other contexts:

- An overlap was observed between *atf3* and *casp3*: at any given time under physiological conditions, 21% of all *atf3*$^{pos}$ cells are also positive for *casp3*. This is of the same order of magnitude as the proportion of *casp3*$^{pos}$ cells in the NSC/NP population overall (15% of all cells are *casp3*$^{pos}$);
- We observed no overlap between *atf3* transcripts and activated Cas3*: in 5 regions of interest from 5 hemispheres, where we could detect 7 Cas3*pos cells, none of which was *atf3*$^{pos}$, and 33 *atf3*$^{pos}$ cells, none of which was Cas3*$^{pos}$.

Thus, under physiological conditions, we did not detect a bias in favor of coexpression at any given time. With so rare events, it is difficult to be statistically more precise. Together, we cannot conclude with certainty. Our observations argue either for Atf3 and Cas3* acting in distinct pathways, or for them being involved in the same pathway, but at successive steps (or with very short overlapping windows of expression). This point is now mentioned in the text (l. 225-229) and the *atf3*/*casp3* double ISH results are illustrated in Fig.S3B. It is further addressed in the discussion (l.368-373) to stress that a direct hierarchy between the two factors in the process uncovered here in adult NSCs remains difficult to ascertain based on our experimental data. It remains that Atf3-L is necessary for the fate bias that characterizes *Cas*$^{Cre}$*Atlas* clones (Fig.5G-K), thus that the two events genetically interact.

2- The timing between atf3 expression and Cas3 activation is especially intriguing as the literature has plenty of papers which suggest that Atf3 itself is necessary for the cleavage of Cas3, as the authors themselves acknowledge at one point. (The fact that no significant change can be observed in delamination events in atf3-L-P2A-nlsGFP or atf3-vivoMO injected animals, seems to suggest an upstream role for the activation of the caspase pathway, but due to the low frequency of such events, such an inference should be treated with due caution.)

We agree that the hierarchical position of Atf3 and Cas3 activities is ambiguous. Published work indicates context- dependent effects, both relative to Atf3 activity upstream or downstream of activated Caspases, and relative to its pro- or anti-apoptotic functions. Here, we show that Atf3 overexpression can induce Cas3* activity in a low number of transduced cells (Fig.5F) while, concomitantly, Atf3 function is needed for the fate-determining action of Cas3* events in NSCs (Fig.5H-K). The latter context relies on a loss-of-function approach, classically considered more reliable, while gain-of-functions can mimic activities of related factors. We now bring this point in the Discussion (l.374).
Regarding delaminations, these are a transient phenotype taking place between the ventricular and parenchymal fates. Thus, the proportion of delaminating cells depends both on the speed of the delamination process and on the behavior of ventricular cells, and is difficult to interpret (in addition to numbers being very low, as mentioned by this Referee). So, we do not believe that this cell population can provide insight into the hierarchical relationship of Atf3 and Cas3*.

3- Is there a reason why clone type sizes are not reported for the irradiation experiments (Figure 7A-D3), like for all the others?

The irradiation experiment that induces Cas3* (Fig.7D-D3) is conducted over 24 hours only. With such a short chase time, no clones of more than one cell are produced. In addition, this experiment was not conducted in the *Cas*$^{Cre}$ *Atlas* background which allows permanent tracing, and Cas3* expression is transient, so counting would not be very rigorous.

4- In animals irradiated with 40 Gy the authors assay cell death 24 hours post irradiation (hpi). Yet, in other experiments - albeit performed in larval animals - after a 25 Gy irradiation a peak in apoptosis can be seen 3.5 hpi (PMID: 26753623). Isn't 24 hpi way too late to observe the effects of the irradiation? Perhaps most Caspase-3 activation due to the irradiation was resolved by this time either by cell death, or by anastasis.

© 2024. Published by The Company of Biologists under the terms of the Creative Commons Attribution License (https://creativecommons.org/licenses/by/4.0/).

In the reference mentioned by this Referee, which is indeed technologically interesting, irradiations are performed in embryos (24 hours post-fertilization), and the cell types considered are not resolved (the promoter driving expression of the FRET sensor is a ubiquitous promoter). As noted by this Referee, it is therefore unclear how this translates to the adult NSC situation. This said, we have now conducted a minimal time series using a higher irradiation dose (80 Gy) and two time points (9 and 24hpi). The induction of Cas3* that we observe in NSCs, as well as the morphology of Cas3*-positive cells, are not significantly different between the two time points, suggesting that working at 24hpi does not lead to an attenuation of the phenotype (at least compared to 9hpi).
These results are now presented in Fig.S5D-E' and text l.292-295, and we added the suggested citation.

5- At the very end of the Results section (lines 331-332) the authors declare that: "At 7dpl, CasCREAtlas fates recorded in lesioned hemispheres did not detectably differ". While the difference is not significant, a very strong trend seems to be present in the reduction of NSCs and increase of single neuron clones in the affected hemipallia of lesioned animals. In this case I think a compelling case can be made for an increase in sample size (especially as 11 hemipallia were used for the controls, but only 3 for the lesioned animals), as it likely could lead to a significant result.

We have redone the lesion analysis to process a similar number of lesioned and unlesioned hemispheres. Indeed, this led to confirm the initial trend, with now a significant increase in neurogenic fates (attached clones, and delaminating and neuronal fates) at the expense of NSCs within *Cas*$^{Cre}$*Atlas* clones (new Fig.7K).
Considering clone types only composed of neurons or neuron progenitors at the time of analysis (delaminating single cells, single neurons and neuron doublets), among which single neurons are largely predominant (>70%), we did not detect a further shift induced by the response to lesion (new Fig.7L). We conclude that, in NSCs that experience a non-apoptotic Cas3* event, lineage termination for neurogenesis is enhanced upon lesion, and that this occurs without a visible change in the neurogenic mode employed. This is now mentioned in l.307-313. We are grateful to our Referee for stimulating this re-analysis.

**Minor remarks:**
1- Single neuron clones should be labelled in yellow, for consistency - see Figure 6H and line 300 in the text.
This has been corrected (Fig.6H and legend).
2- Expressions with Latin origin, such as in situ and in vivo should be always italicized.
This has been corrected.
3- For consistency, “Ventricular" should start with a capital letter in Figures 5D,E.
This has been corrected.
4- In Figure S4C radiation strength and chase time are missing.
This has been corrected (now Fig.S5C).
5- The reference for the Morizet et al. preprint is incomplete.
This has been corrected.

**Reviewer 2**
**Advance Summary and Potential Significance to Field:**
This work identifies molecular mechanisms underlying the direct conversion of neural stem cells (NSCs) in the adult zebrafish brain, by focusing on the potential role of non-apoptotic caspase activation in cell fate. To do so, the authors explore caspase activation events that do not result in apoptosis and the role of the transcription factor atf3, which is a known mediator of non-apoptotic caspase activation events in other species. The work, therefore, addresses a poorly understood and interesting process by proposing that processes of caspase activation not resulting in apoptosis may have an effect in cell fate decisions.

**Comments for the Author:**
This work identifies molecular mechanisms underlying the direct conversion of neural stem cells (NSCs) in the adult zebrafish brain, by focusing on the potential role of non-apoptotic caspase activation in cell fate. To do so, the authors explore caspase activation events that do not result

© 2024. Published by The Company of Biologists under the terms of the Creative Commons Attribution License (https://creativecommons.org/licenses/by/4.0/).

in apoptosis and the role of the transcription factor atf3, which is a known mediator of non-apoptotic caspase activation events in other species. The work, therefore, addresses a poorly understood and interesting process by proposing that processes of caspase activation not resulting in apoptosis may have an effect in cell fate decisions. The authors have developed, over the last few years, very innovative and rigorous in vivo imaging strategies by using reporters and live microscopy, followed by quantitative analysis, to analyze adult NSC behavior in the fish pallium at the single cell level, positioning their studies at the forefront of those devoted to cell fate decisions in adult neurogenesis. Previous studies by the same group have demonstrated the process of NSC direct conversion, meaning the direct differentiation of NSCs into neurons without previous cell division. This has not been conclusively proven in mammals, despite some data in the dentate gyrus, but many similarities between zebrafish and rodent adult NSCs make these studies relevant and offer new hypotheses to be tested in the rodent brain.

The authors first rigorously test the strains of transgenic fish that are required to address the hypothesis. To do so, they characterize NSC direct conversion in a strain expressing a marker of apical surfaces (ZO1-mKate2) and a marker of neurogenic fate (delta) and cleverly use experimental timelines and cell dimensions to focus on the cell dynamics of NSCs vs. other neural progenitor (NP) cells in direct conversion to a neuronal fate without cell division. To detect caspase activation they make use of a fish strain (Cas-cre Atlas) that expresses, under the control of an NSC promoter (her4), a cre recombinase targeted to the cell membrane and whose release from the plasma membrane is dependent on a caspase target peptide (DEVD). This reporter line, which is sensitive to caspase activation is also thoroughly tested in the manuscript. A control line expressing a target peptide for other proteases is used as a control. The authors elegantly use lineage tracing (from the Cas-cre Atlas line and a transgenic line for conditional cre induction in NSCs) and BrdU pulse-chase experiments combined with their experience in imaging direct conversions and describe that caspase-mediated effects correlate with a bias of NSC fate towards direct neuronal differentiation. Although a causal relationship cannot not be demonstrated, because the caspase activation is transient but the recombination is stable, and furthermore some activations may lead to cell death, the association is sufficiently strong. Furthermore, induction of caspase activation by apoptotic inducer camptothecin results in direct conversion.

With the proposal that the observations could be the result of the cleavage of signaling pathways effectors by caspases, they focus the second part of their work on atf3. The authors show that only a few delaminating NSCs in the adult pallium express a long form of atf3 mRNA. Loss- and gain-of-function experiments demonstrate that atf3 regulates direct conversion. The authors then analyze direct conversion in conditions of apoptosis induction by irradiation and by mechanical lesion to the pallium. Although the former does not induce the process, despite lesions in the DNA, the latter results in more atf3-positive NSCs and delaminating cells.

The work is relevant and well executed.

**Some recommendations for improvement are proposed.**

1- Regarding experiments, the authors should better connect caspase activation with atf3. One suggestion would be to use camptothecin in Cas-cre Atlas adults injected with the morpholino (MO) against atf3-L. This is especially relevant as they show in Figure 7 that a mechanical injury increases the numbers of cells expressing atf3-L but this could be a result of the lesion independent of the caspase activation.

We attempted the suggested experiment, but the fish poorly survived the consecutive intracranial injections of CPT and MO, perhaps all the more as we felt that several CPT injections were necessary to cover the 5-7 days needed for a tractable effect of the MO. This very high mortality made us doubt of the validity of the few survivors.

As a further attempt to link Atf3 and Cas3*, we used various schemes of sensitive smFISH (or smFISH and IHC), as detailed in our response to Referee 1, point 1, to compare the expression of the two factors. An overlap was detected in a minority of cells for the coexpression of *atf3* and *casp3*, although this is not enriched over the expected intersection of individual expression patterns. Together, with the current tools (an Atf3 antibody may help, but is not available for zebrafish), we cannot conclude with certainty. Our observations argue either for Atf3 and Cas3*

© 2024. Published by The Company of Biologists under the terms of the Creative Commons Attribution License (https://creativecommons.org/licenses/by/4.0/).

acting in distinct pathways, or for them being involved in the same pathway, but at successive steps (or with very short overlapping windows of expression). This point is now mentioned in the text (l. 225-229) and the *atf3/casp3* double ISH results are illustrated in Fig.S3B.

At this point, we feel that the experiment performed under physiological conditions showing that blocking Atf3 reverts the fate of *Cas*$^{Cre}$ *Atlas* clones is the most compelling to support the fact that Atf3 is necessary for the expression of the *Cas*$^{Cre}$ *Atlas* fate, although we indeed cannot conclude that Atf3 and Cas3* are hierarchically connected in the same pathway.
Our discussion paragraph addressing the “link between Cas3*/Cas7* and Atf3” as a “key missing step” (l.367-383) has been expanded to provide, we hope, a fair account of the data and their interpretation.

2- Why are the experiments of overexpression of atf3 and MO shown in Figure 5 performed differently? They need to homogenize these data.

2 days of Atf3-L overexpression are sufficient to trigger a highly significant NSC behavior phenotype, which is why this timeframe was used to demonstrate the gain-of-function effects and to compare Atf3-L and Atf3-S (Fig.5D,E). The other experiments (Fig.5F-K) address the effect of Atf3 gain- and loss-of-function on *Cas*$^{Cre}$ *Atlas fates*, and this reporter system requires several days for Hmg2bmCherry to be detectable. In addition, our previous work demonstrated that MOs are most active at around d3 post-administration. For these reasons combined, we tracked fates after 7 days.
To make the analyses more easily comparable, though, we have now homogenized our clonal categories to be the same in Fig.5D, E and K.

3- The results in experiments shown in Figure 7E-K are not clearly stated. As explained in the text, it appears that more delaminating cells expressing atf3 are observed in the lesioned hemisphere at 3 days post lesion, but this is not quantitated. The quantitation is shown at 7 days post lesion but no significant differences are found (Figure 7K). That together with a rather unclear description in the text does not clarify whether the mechanical lesion truly induces direct conversion for repair. If this is so, the authors could try to see if atf3 MOs interfere with the repair response.

As also suggested by Referee 1, we have redone the lesion experiments in *Cas*$^{Cre}$ *Atlas* adult fish with a comparable number of lesioned and unlesioned brains. We can confirm the initial trend with now a significant increase in neurogenic fates (attached clones, and delaminating and neuronal fates) at the expense of NSCs within *Cas*$^{Cre}$*Atlas* clones (new Fig.7K). Considering clone types only composed of neurons or neuron progenitors at the time of analysis (delaminating single cells, single neurons and neuron doublets), among which single neurons are largely predominant (>70%), we did not detect a further shift induced by the response to lesion (new Fig.7L). We conclude that, in NSCs that experience a non-apoptotic Cas3* event, lineage termination towards neurogenesis is enhanced upon lesion, and that this occurs without a visible change in the neurogenic mode employed. This is now mentioned in l.307-313; we have re-written this paragraph to increase clarity.

We did not quantify the increase in *atf3* expression for two reasons: the number of positive cells upon lesion makes it virtually impossible to count, and this high number of cells also precludes grouping positive cells into clonal events. For a similar reason, we cannot quantify the absolute number of *Cas*$^{Cre}$*Atlas* clones. Indeed, we can only record clones that are sufficiently far apart from each other to be unambiguously identified as clones. Thus, we cannot formally tell whether and by how much *Cas*$^{Cre}$*Atlas* events are actually induced by lesion.
Overall, the analysis of lesions and the relationship between Atf3 and Cas3* in this context would require a full project, which we feel goes beyond the scope of this paper, while we convincingly demonstrate that Atf3 is required for the bias towards neurogenic lineage termination in *Cas3*$^{Cre}$*Atlas* events under physiological conditions (Fig.5H,K). We now directly mention that the lesion analysis does not aim to directly link Atf3 and Cas3* (l.313).

© 2024. Published by The Company of Biologists under the terms of the Creative Commons Attribution License (https://creativecommons.org/licenses/by/4.0/).

4- The authors need to improve the way they explain the experiments, especially for those who do not work in zebrafish. For example, they need to include a clear timeline for the development of zebrafish and how the different experiments relate to this timeline. That could be done in coordination with the schemes used in the different figures (for example, Figure 2 and 3). In addition, they should always use the same timings (for example, mpf and wpf are used in different parts of the manuscript).

We have added a general timeline along which we positioned all the experiments of the paper (new Fig.2A). We also restricted the mention of time points post-fertilization to days (dpf) and months (mpf), avoiding weeks.

5- Also, please try to homogenize the graphs (Figures 3D, 5D, K, and 7K). Furthermore, in these graphs, the attached and detached categories include the other ones (Single Neu, Neu doublet, NSC clones Single NSCs) and therefore should be represented in different graphs for clarity. Also, try to use the same order and categories throughout the graphs, especially as they need to be compared.

All graphs have been modified so that the categories represented are exclusive. We also ordered the categories consistently. We did our best to uniformize the data (in subdivisions of three categories, for example, while further subdivisions are shown in Supp. data). But it was not possible to fully uniformize the data, notably given that the type of clones observed depends on the chase time of the experiment: for example, after labeling individual NSCs (whether with an electroporated plasmid or a Cre-lox), tracking for 2 days mostly generates singlets, doublets or delaminating cells, while tracking for one month can generated larger clones.

6- The authors need to name the atf3 forms (short and long) with their nomenclature before they use them in the electroporation experiments so the latter can be more easily followed.

This has been added (l.230-231).

**Minor things:**
- In Figure 1A: add the symbols to the figure instead of just in the figure legend. Also, it would be clearer if all the cells, delaminating or not, would be entirely delineated.

This has been done.
- In Figure 3, I would suggest using the same representation of the experimental design as in Figure 2 to ease reading.

This has been done (see Fig.3A, we have now visually illustrated the genotypes as in Fig.2B)
- In Figure 3A and related text, better define “induced” and “scored” clones.

In Fig.3A, we have now indicated “4-OHT-induced clones” and replaced “scored clones” with “quantified clones”. We also added these precisions to the text (l.189 and beyond)
- Some sentences that are difficult to understand are found in lines # 182, 270, 320, and 331.

We have split and/or simplified these sentences. L.331 referred to the paragraph on lesions and this paragraph has not been re-written (l.307-313).
- In line 246 an “and” between “fluorescent” and “chromogenic” is missing.

Actually, here we used Fast red, which is both chromogenic and fluorescent. We have not changed our sentence but specified “Fast red” in the legend of the figure for clarity.
- Also in line 246, the ISH is used to address not only the “morphology”, but also the “location” of atf3-positive cells.

Yes indeed. We have added this (now l. 222).

**Reviewer 3**
**Advance Summary and Potential Significance to Field:**
This study explores the role of a novel pathway on the process of neuronal delamination and direct neurogenesis in the adult zebrafish brain. Specifically, the authors investigate the potential involvement of the caspase non- apoptotic pathway in neuronal delamination and direct, offering a new perspective on this phenomenon. Using Caspase reporter lines, the study aims to establish a correlation between caspase non-apoptotic activation and the occurrences of direct neurogenesis. The findings reveal an increase in single neuron clones upon Caspase activation, indicative of an increase in neuronal delamination. this study also examines the effects of caspase non- apoptotic

© 2024. Published by The Company of Biologists under the terms of the Creative Commons Attribution License (https://creativecommons.org/licenses/by/4.0/).

activation and neuronal displacements following injury, although the overall outcome of this approach is written is a convoluted way. In addition this study explores the role of atf3, a mediator of non-apoptotic events, on direct neurogenesis through overexpression and knockdown (KD) experiments. The data suggest that Atf3 KD diminishes neuronal delamination, whereas overexpression amplifies these events. Overall, this study stands out for its innovative approach to manipulating adult neurogenesis, and exploring a novel pathway on the process- a task typically full of challenges.

**Comments for the Author:**

1- The flow of the manuscript could be improved; the sequence of experiments better justified. Consistent use of nomenclature, such as NSC or NPs, throughout the manuscript would also contribute to clarity. The link between the initial morphometric analysis and subsequent sections of the study would also help improved the flow of information.

We have now modified the Introduction (l.60-66 and 83-84) to announce the reasoning underlying the sequence of experiments that we performed: (i) first, using intravital imaging, a quantitative analysis of the morphodynamic parameters of direct neuronal conversion, with the hope to identify predictive parameters that could lead us to a mechanism; (ii) second, because this turned out to be negative, an educated guess focusing on non-apoptotic caspase events, which in other instances had been correlated with cell fate choices. We also directly mention the reason for this change of focus in the text (l.143-144). We agree with our Referee that presenting the exact reasoning (and failures) will increase clarity. It remains that our findings, due to technical limitations, do not directly link the different processes studied (delamination proper, *Cas*$^{Cre}$*Atlas* events, Atf3 function). We consider them to be a first discovery of convergent information, that will need to be deepened in future work.

Regarding the nomenclature issue (NSCs or NPs), upon re-reading our text we realized that it was due to the use of “NPs” when referring to *Cas*$^{Cre}$*Atlas* events in the embryonic and larval pallium. This introduced an ambiguity also on the specificity of the *her4* promoter (see minor point 1 below as well). Embryonic and larval radial glia are classically referred to as NPs (and not NSCs) only because we cannot ascertain that all of them will be maintained until adulthood. But they are the most upstream cells of the neurogenic lineage, akin to adult NSCs. For clarity, we now use the term “radial glia” at embryonic and larval stages (l.161).

2- Text related to Fig1 and FigS1 should be simplified and consistent to help the reader's understanding. Gfap:ZO-1-mKate labels the apical surface of NSCs but not NPs. However, this distinction is not always clear in the text and FigS1, leading to confusion regarding the identification of NPs solely by ZO1-mKate expression. To improve clarity, consistent labelling and clear definitions would be helpful. For instance, the apical surface of NPs is defined by Zo1 immunofluorescence (+) Zo1-mKate (-) expression, while that of NSCs can be identified by Zo1 immunofluorescence (+) Zo1-mKate (+) or gfap:tomato+ and Zo1 immunofluorescence (+). It would also be beneficial to provide an example of an isolated NP for clarification in FigS1.

We have modified the text as suggested, listing the exact readouts used for NSCs vs NPs, in the case of adjacent NPs and in the case of isolated NPs (l.126 and beyond) (in fact using Tg(*gfap:gfp*) fish to count isolated NPs, this was a mistake in the first version).

We also added a panel showing examples of isolated NP, identified as a Zo1$^{pos}$, *gfap:GFP*$^{neg}$ surface by IHC in the Tg(*gfap:gfp*) transgenic background (new Fig.S1C).

3- A more detailed explanation of statistical treatment would be helpful and the number of fish per experiment should be provided. I would advise to ask a statistician to discuss the choice of statistical tests. Chi-square test tend to indicate significant differences between control and treated groups and demonstrate that observed differences are not solely due to data variability, they do not allow for the interpretation of whether the proportion of lineage type 1 is significantly increased or decreased in control compared to a treated condition. Therefore, it is confusing that most graphics appear to compare averages between control and treated conditions as if an ANOVA was applied. However the figure legends mention Chi-square. Authors should try to improve this

© 2024. Published by The Company of Biologists under the terms of the Creative Commons Attribution License (https://creativecommons.org/licenses/by/4.0/). 

important part of the manuscript as this would help draw the appropriate conclusions.

We have now entirely redone the statistical analyses with help of a statistician from the Institut Pasteur Bioinformatics Hub. It was indeed our mistake to have indicated the results of individual Fisher's exact tests onto the graphs generated from the contingency analysis using a global Chi-square test. What we now display is: (i) a Chi-square analysis testing for global differences between experimental and control conditions with the resulting value, and, in cases where a statistically significant global difference is observed, (ii) the p-values corresponding to the comparisons between individual fates, using a Fisher's exact test and Bonferronni correction. The latter p- values are no longer indicated on the graphs but below the panels, and in the figure legends.
The number of fish per experiments is always provided. What we record is the number of hemispheres, taking only one hemisphere per fish.
We now specify these points in the Materials and Methods section on statistics (l.503-510).
We also needed to consider one comment of Referee 2 asking us to use, as much as possible, comparable categories between the different experiments. This led us to group some fates, in a biologically logical manner. Together, our conclusions remain unchanged, with one exception, which is that we cannot sort between the different "neuron-only" fates (1N, 2N or more) in the long-term analysis of CasCreAtlas fates (Fig.3D and Fig.S2B). In this part, we now conclude that NSCs that experience a non-apoptotic Cas3*/Cas7* event are subject to a statistically significant bias towards lineage termination, within which the generation of single neurons is largely predominant (>75% of cases) (l.210).

**Minor corrections and suggestions**

1- Clarify whether the gfap promoter drives expression in NSCs and if her4 drives expression in NPs and NSCs.

Both promoter fragments actually drive expression in the same cells (NSCs), as published in Than-Trong et al., 2020. This information was indeed missing and we have now added it (l.152).

2- Inconsistencies in the text regarding her4 association with both NSCs and NPs should be addressed.

Indeed our text was ambiguous. We re-wrote the introduction section on this point (l. 45-48).

3- Additionally, on page 5, Fig.S1B should be corrected to S1C and Fig.S1C to S1D.

This has been corrected.

4- In FigS1B, I think "1 cell" should be replaced with "1 NSC" as in FigS1D, and ">1 cell (doublets or triplets)" should be replaced with "NPs."

Indeed ">1 cell" corresponds to NPs, and we have now added this on the figure. However, "1 cell" may be "1 NSC" or "1 NP" (in the latter case, falling into the category studied in panel 1C). So in that case we have preferred to leave the legend as "1 cell".

5- Consider removing "N. Dray unpub." and briefly discussing Baek et al., 2018.

This has been done (l.332).

6- Define "Tp" in the main text.

This is done in the introduction (l.56). We added it again in the results (l.95).

7- Rectify misaligned diagrams in some of the figures.

This probably referred to the schematics of clone types above diagrams. They have been aligned.

---

Second decision letter

MS ID#: dev.204381

MS TITLE: Non-apoptotic caspase events and Atf3 expression underlie direct neuronal differentiation of adult neural stem cells

AUTHORS: Laure Bally-Cuif, Frédéric Rosa, Nicolas Dray, Sébastien Bedu

© 2024. Published by The Company of Biologists under the terms of the Creative Commons Attribution License (https://creativecommons.org/licenses/by/4.0/).

You will be pleased to know that the referees are happy with your revisions and there are just a couple of minor suggestions/corrections to make prior to publication. The referees' comments are appended below

Reviewer 1

*Advance summary and potential significance to field*

This work identifies molecular mechanisms underlying the direct conversion without division of neural stem cells into neurons in the adult zebrafish brain, a poorly understood process that could also be happening in other vertebrates including mammals.

Comments for the Author

The authors have adequately addressed my concerns by conducting additional experiments or providing further clarifications.

Reviewer 2

*Advance summary and potential significance to field*

In their submitted manuscript Rosa and co-workers present a compelling case for a hitherto unrecognized role of anastasis in the neurogenesis of the zebrafish brain. In the manuscript they reanalyse earlier datasets from the Bally-Cuif lab and also develop an innovative new genetic tool, the CasCRE Atlas to correlate delamination events observed in the ventricular, neurogenic zone of the zebrafish pallium, with the occasional activation of Caspase3 in the same cells. I do believe, that the revised version of the manuscript is suitable for publication in Development, I have only minor suggestions, see below.

Comments for the Author

1. The authors state (ln 225) that they could not detect atf3 and cas3 co-expression in their RNAscope dataset, however Figure S3B actually does show co-expression of these two markers. Even if such events are rare, they still might be important for the suggested mechanism. (Related suggestion in the text the authors use cas3 as an abbreviation, whereas on the figure it is casp3 - this should be uniform).

2. The authors should also make a comment in the manuscript whether they do see co-expression of the two genes in the Moritz et al. in silico dataset, or not.

3. It is also not made clear if the in silico dataset captures both isoforms - I suppose it does, but perhaps it should be made clear.

4. Ln 353 - in silico should be in italics

5. Ln 462 - hybridization should not be in italics.

Reviewer 3

*Advance summary and potential significance to field*

The authors addressed my concerns and in the current shape the manuscript reads nicely.

---

**Second revision**

© 2024. Published by The Company of Biologists under the terms of the Creative Commons Attribution License (https://creativecommons.org/licenses/by/4.0/). 

Author response to reviewers' comments

**Response to Referee 2**

1. The authors state (ln 225) that they could not detect atf3 and cas3 co-expression in their RNAscope dataset, however Figure S3B actually does show co-expression of these two markers. Even if such events are rare, they still might be important for the suggested mechanism. (Related suggestion in the text the authors use cas3 as an abbreviation, whereas on the figure it is casp3 - this should be uniform).

2. The authors should also make a comment in the manuscript whether they do see co-expression of the two genes in the Moritz et al. in silico dataset, or not.

3. It is also not made clear if the in silico dataset captures both isoforms - I suppose it does, but perhaps it should be made clear.

→ Comments 1-3 are now addressed together on l.225-228 in a paragraph that reads:

"Of the two *caps3a* and *b* duplicates, only *casp3a* expression was detected in scRNAseq (Fig.S3A), and only 5% of *casp3a*$^{pos}$ cells were *atf3*$^{pos}$ in this dataset, equivalent to the overall proportion of *atf3*$^{pos}$ cells among all NSCs (Morizet et al., 2024). In situ, we only detected rare instances of coexpression of *atf3* and *casp3a* (using RNAscope Hiplex whole-mount ISH) (Fig.S3B) or *atf3* and Cas3* (revealed by IHC) (not shown) under physiological conditions".

We have also modified abbreviations to be consistent (*casp3a* everywhere).

4. Ln 353 - in silico should be in italics

This has been corrected.

5. Ln 462 - hybridization should not be in italics.

This has been corrected.

---

Third decision letter

MS ID#: dev.204381

MS TITLE: Non-apoptotic caspase events and Atf3 expression underlie direct neuronal differentiation of adult neural stem cells

AUTHORS: Laure Bally-Cuif, Frédéric Rosa, Nicolas Dray, Sébastien Bedu

I am happy to tell you that your manuscript has been accepted for publication in Development, pending our standard publication integrity checks.

© 2024. Published by The Company of Biologists under the terms of the Creative Commons Attribution License (https://creativecommons.org/licenses/by/4.0/).
